# Interactive Light Stimulus Generation with High Performance Real-Time Image Processing and Simple Scripting

**DOI:** 10.3389/fninf.2017.00070

**Published:** 2017-12-13

**Authors:** László Szécsi, Ágota Kacsó, Günther Zeck, Péter Hantz

**Affiliations:** ^1^Computer Graphics Research Group, Budapest University of Technology and Economics, Budapest, Hungary; ^2^Neurochip Research Group, Natural and Medical Sciences Institute at the University of Tübingen, Reutlingen, Germany; ^3^Department of Laboratory Medicine, University of Pécs, Pécs, Hungary

**Keywords:** light stimulus, patterned illumination, video processing, retina, psychophysics, GPU

## Abstract

Light stimulation with precise and complex spatial and temporal modulation is demanded by a series of research fields like visual neuroscience, optogenetics, ophthalmology, and visual psychophysics. We developed a user-friendly and flexible stimulus generating framework (GEARS GPU-based Eye And Retina Stimulation Software), which offers access to GPU computing power, and allows interactive modification of stimulus parameters during experiments. Furthermore, it has built-in support for driving external equipment, as well as for synchronization tasks, via USB ports. The use of GEARS does not require elaborate programming skills. The necessary scripting is visually aided by an intuitive interface, while the details of the underlying software and hardware components remain hidden. Internally, the software is a C++/Python hybrid using OpenGL graphics. Computations are performed on the GPU, and are defined in the GLSL shading language. However, all GPU settings, including the GPU shader programs, are automatically generated by GEARS. This is configured through a method encountered in game programming, which allows high flexibility: stimuli are straightforwardly composed using a broad library of basic components. Stimulus rendering is implemented solely in C++, therefore intermediary libraries for interfacing could be omitted. This enables the program to perform computationally demanding tasks like en-masse random number generation or real-time image processing by local and global operations.

## 1. Introduction

Investigating light-sensitive tissues, analyzing the optic tract or doing experiments in visual psychophysics require light stimulation with complex spatio-temporal modulation. The growing demand is fulfilled by several free and commercial light stimulus-generating software, having different benefits and disadvantages. The most notable of these are the Psychtoolbox, PsychoPy, VisionEgg, OpenSesame, E-Prime and Presentation (Yoonessi and Yoonessi, 2011; Strasburger, 2015; Software Comparision, 2016).

Psychtoolbox (Lu and Dosher, 2013; Psychtoolbox, 2016) is a package of interface routines between Matlab and OpenGL. It also handles computer hardware like graphic cards, sound devices, and mechanical feedback tools. It has a large user community, and offers most of the presently used light and sound stimuli. In order to run it at its full potential, commercial software (Matlab) has to be installed on the computer. Matlab is available for all common operating systems (Windows, Linux, and Mac).

PsychoPy (Peirce, 2009; Kubilius, 2014; Psychopy, 2015) is freeware, platform-independent application to allow the presentation of light and sound stimuli. It uses the Pyglet library to interface between Python and OpenGL, and consists of about 20 submodules which can be used as building blocks to create visual and sound stimuli. The interpreted Python code redirects computationally intensive tasks to C and FORTRAN via the Numpy module of Python. PsychoPy can also interact with external hardware. Beside traditional programming, it offers a GUI as well.

VisionEgg (Straw, 2008; VisionEgg, 2009; Muller et al., 2015) is another freeware cross-platform library to generate visual stimuli. OpenGL commands are accessed through the PyGame multimedia modules. Particular attention is given to luminance and temporal calibration. Several interfacing techniques to input devices such as mice, or digital triggers are also supported. In contrast to PsychoPy, it uses a strongly object-oriented model of programming. However, for users who are less experienced in software development, it is hard to understand object-oriented frameworks.

OpenSesame (Mathôt et al., 2012; OpenSesame, 2016) is a free graphical experiment builder software compatible with all common operating systems. It has comprehensive and intuitive GUI, but also allows Python scripting for more complex tasks. The software supports various external devices. Although it just offers a small number (about 20) of functions for implementing visual stimuli, it is compatible with PsychoPy routines. Therefore, the same considerations as for PsychoPy apply.

E-Prime (Spapé et al., 2014; E-Prime2, 2016) and Presentation (Presentation, 2016) are commercial software available only for the Windows operating system. They offer GUI and custom scripting language for assembling visual stimulation experiments, with building elements covering the most common patterns. Both software can interface with external hardware.

The ViSaGe MKII Stimulus Generator and the corresponding CRS Toolbox Matlab package represents a system consisting of dedicated hardware and corresponding software. This solution, offering wide functionality, has been developed by the Cambridge Research Systems (DepthQ 360 DLP Projector, 2016). However, purchasing expensive hardware and commercial software is required to run the system.

A public domain software which enables straightforward stimulus development without prerequisites in programming skills, is flexible enough to meet the demands of various fields, offers interactivity, and can perform computationally demanding tasks like real-time video processing, is still in demand. Our software, GEARS (www.gears.vision), is a standalone freeware solution, based on a new computational workflow model. Although most common requirements can be addressed by the software listed above, none of them is optimized for computationally intensive tasks and interactivity. In classic packages, computations are executed by a small number of CPU processors, while hundreds of GPU processor cores that are present on an average graphics card, and could run the task in parallel, are not utilized. By taking advantage of the parallel architecture of GPUs for non-graphical tasks, GEARS is able to perform real-time operations like tone mapping, histogram equalization or contrast stretching, filtering operations like edge enhancement by difference-of-Gaussian kernels, image sharpening, as well as further operations in real or Fourier space (Klette, 2014). If a large batch of random numbers is required in every frame, GPU-based parallel random number generation is also possible. Only some of the previously mentioned packages can perform these tasks in real-time, and not without challenges to the user and the hardware. Pre-computing stimuli, recording and playing them back as video streams could be used for computation-intensive cases. However, such a solution has a series of drawbacks. Facilitating interactivity is obviously not possible. Video compression artifacts may be prohibitive for some artificial stimuli, and handling uncompressed high resolution footage carries storage and bandwidth requirements not widely available. Decoding high resolution video may not be possible at or above 60 frames per second. Moreover, it is extremely difficult (and conflicts with frame rate requirements) to implement synchronization with external electronic signals marking time points, which is of pivotal importance for physiology experiments.

In order to illustrate the advantage of GPU-based parallelization, let us consider a two-dimensional Fast Fourier Transformation (FFT) of a 1024 × 1024 image. This is an operation that needs to be performed twice in every frame to realize real-time convolution with large kernels. At 60 Hz, 0.016 s are available to compute a frame. On a laptop computer with an Intel I5 processor, we measured 0.08 s for CPU execution, which is prohibitively large. However, using an nVidia GeForce Titan GPU with 3072 GPU cores, the computation is performed in 0.002 s, i.e., in 40 times less time than the CPU solution. If further processing of the data also happens on the GPU (as in GEARS), we do not have to consider the time cost of transferring the input and the output between the CPU and the GPU.

The new workflow model of GEARS is more inclusive than any of the software mentioned above. Previous solutions adhered to the classic image synthesis method of drawing polygons by filling image pixels. Therefore, variation of pixel color within shapes could only be achieved using precomputed textures. Our model exploits the full computational capabilities of the GPU as it does not rely on polygon rendering, but evaluates formulas that describe shapes, spatial and temporal patterns for every pixel. This provides more flexibility, and allows a framework where individual aspects can be combined freely without writing new programs.

Thus, compared to previous packages, GEARS offers a much broader selection of components, at multiple levels of granularity, to build stimulus sequences. Executable spatio-temporal light patterns, namely the *stimuli*, are used to build up diverse *stimulus sequences*. Similar to computer game engines, elements of the rendering software underlying the *stimuli* can be assembled using a component-based system, which consists of a large number of elementary building blocks referred to as *stimulus building components* (SBC). SBCs are written in Python, but they configure a rendering backend implemented solely in C++. Only the C++ engine has to access the GPU hardware. Thus, GEARS does not require intermediary libraries for PythonOpenGL interfacing. These solutions endow GEARS with unprecedented flexibility and computational power. Moreover, real-time, interactive changing of stimulus features via simple feedback mediated by mouse, keyboard, or a microcontroller is also possible, even during computationally demanding applications.

Since GEARS represents a radically new framework for computationally demanding stimulus generation, with some elements coming from high-performance computer game software, features present in already existing software (PsychoPy and VisionEgg in particular) had to be re-created. GEARS includes hundreds of scripts (Python programs interpreted by GEARS in runtime) implementing mainstream stimulus elements for physiology and psychophysics that can easily be modified and combined.

In most light stimulus software developed up to this time, simple usage and flexibility were conflicting demands. A graphical user interface (GUI) has inherent limitations and requires permanent development. In contrast, if customizing the software is implemented through an application programming interface (API), the user has to possess deep programming skills.

In order to avoid drawbacks of application programming interfaces (APIs) and graphical user interfaces (GUIs), we have developed a solution that we call the visually aided scripting interface, or VSI. This is an integrated, Python-based script editor to create and configure stimulus sequences. This combines the potential of Python programming with an entry-level user experience that is not more challenging than setting parameters on a GUI.

In order to correlate experimental data (like voltage traces) with stimulus events, timing information of the stimuli has to be recorded as well. GEARS offers high precision signaling of significant temporal markers like start and termination of relevant stimulus elements, or presentation of individual frames. Driving external devices like shutters, filters, or monochromators can also be required: for this purpose, stimulus definitions include strategies for emitting TTL signals via USB ports equipped with RS232/TTL adapters. The software warns the user if the computational demand exceeds computer power, and adjustments to performance-critical stimulus parameters are needed. It is also possible to prepare the frames of a stimulus or stimulus sequence in advance, and project it as a plain video during the experiment—but this may pose comparably steep hardware requirements for high frame rates, resolution, and fidelity. Control of external devices is possible through their runtime library interfaces.

The software has been developed in a teamwork with retina physiologists and psychophysicists. Demands of experimental scientists have been implemented in GEARS. Presently GEARS runs on the Windows platform, but most of its code base is platform-independent, relying on Python and OpenGL. Implementation of performance-critical, platform-dependent window management and event handling functionality for the Linux operating system is in progress.

## 2. Software operation

**Figure 1:**
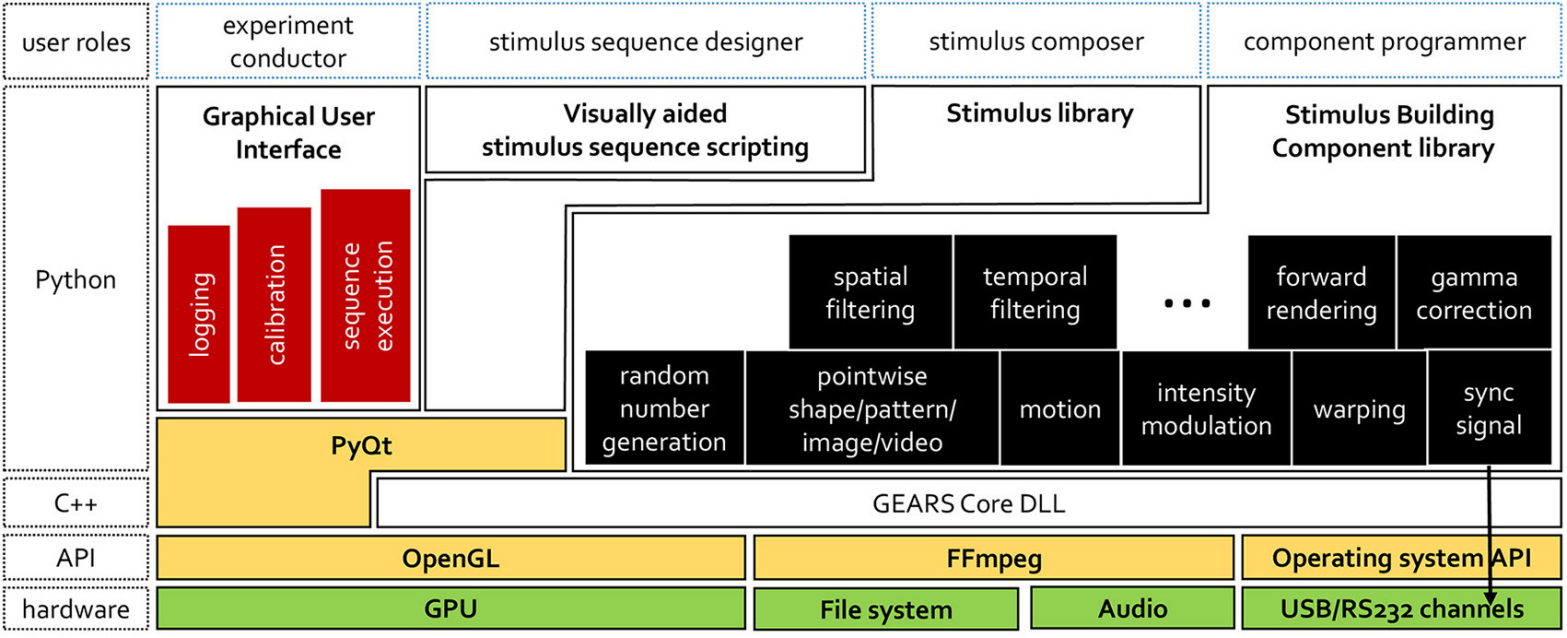
The GEARS software stack. Stimulus sequences can be executed using the GEARS GUI. New sequences can be composed with a built-in visually aided script editor. A library of ready-to-use, parametrizable stimuli and stimulus sequence scripts are provided. Custom stimuli can be assembled from stimulus building components.

GEARS is a C++/Python hybrid using OpenGL graphics, that generates GLSL shaders dynamically. Figure 1 shows the software stack. Stimulus rendering is implemented solely in C++, while Python is used for user interface and scripting. Thus, it is the role of the Python layer (detailed in section 2.1) to assemble stimulus sequences via calls to the C++ layer. Afterwards, stimulus sequence display runs natively, with direct operating system calls. We do not have to use intermediary libraries for window management or Python-OpenGL interfacing (e.g., PyQt, PyOpenGL, and PyGame). This allows us to sidestep overhead or limitations of existing APIs, e.g., for combining video rendering with OpenGL.

### 2.1. Scripting model

GEARS offers a comprehensive library of mainstream stimulus sequences prevalent in retina science. These are implemented by *stimulus sequence scripts*, which are intuitive Python scripts consisting of concatenated stimuli with their parametrizations. For example, a stimulus sequence may consist of animations of bars progressing in different directions, with uniform gray fullfield illuminations in between. A large set of pre-fabricated stimuli is available in GEARS. For example, a moving bar stimulus can be parametrized by the dimensions, sweeping speed and direction, among others. The scripts defining the stimuli themselves consist of simple and intuitively parametrizable stimulus building components (SBCs), which control a certain feature of the stimulus. For example, the moving bar stimulus combines a rectangle shape SBC with a sweeping motion SBC, while a stimulus showing a spot with varying intensity is composed using a shape SBC and a modulation SBC.

New, custom stimuli can also be assembled using the same mechanism, applying an arbitrary number of SBCs. This does not require the user to edit the SBC implementations that connect to the C++ layer or define functions in the GLSL shading language. The user has to deal solely with the interface of SBCs for intuitive parametrization.

### 2.2. Stimulus sequence control GUI and visually aided script editing

GEARS is equipped with a GUI developed for the design, configuration, tone mapping calibration, overview, and execution of stimulus sequences. The stimulus execution screen shows the sequence overview with sample frames, and/or timeline plots of important temporal characteristics (e.g., modulation intensity or synchronization signals).

**Figure 2:**
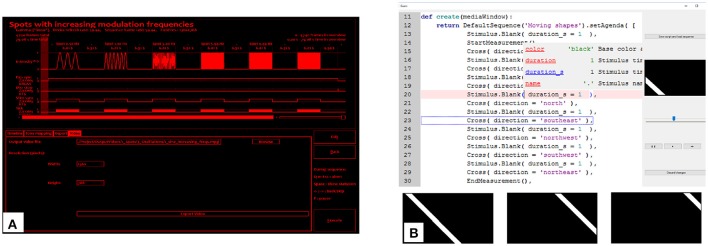
The **(A)** sequence overview and **(B)** visually aided script editor of GEARS. Editable Python code of the stimulus sequence script is listed. Call tips displaying currently edited SBC parameters with their default values and descriptions are shown automatically. A seekable dynamic preview of the stimulus is also displayed while marking the part of the script under execution.

The authoring of the stimulus sequence scripts using intuitively parametrizable stimuli, as well as composing custom stimuli from SBCs, is supported by a visually aided scripting interface, or VSI (Figure 2). Although the scripts defining the stimulus sequences are valid Python code, they were designed to be concise and well readable even for complex stimuli. In practice, for a typical user of GEARS, it is sufficient to list and parametrize pre-made stimuli, accompanied by elementary flow control commands like *for* loops. Even authoring of new stimuli can be performed without low-level programming skills, as our component-based model allows fast and flexible stimulus assembly using SBCs. Both the components and the stimulus classes already assembled are highly customizable, with numerous parameters. GEARS offers more than a hundred of different SBCs and dozens of stimuli implemented, and all of them have their own parameter interfaces. Users are not expected to keep them all in mind, since the VSI of GEARS incorporates a customized Integrated Development Environment (IDE). This solution is based on the QScintilla (Hodgson, 2016) source code editor, but enhanced to specifically support SBC interfaces, and synergize with an instant visual representation of the edited components, stimuli, and the stimulus sequence script. Customized code completion and call tips provide instant documentation, as well as clickable, floating listings of possible components and parameters.

The VSI offers side-by-side visual presentation of the editable features of the stimulus elements. Moreover, it shows the time flow of the stimulus sequence prepared for execution. The scripts can be saved and recalled straightforwardly.

The creation of new SBCs for innovative stimuli is not in the scope of the VSI, but it is a fairly simple Python/GLSL programming task that typically only requires changing some formulae in existing program code.

For further details on GUI, we refer to Supplementary section 5, to the user's manual, and to the homepage of GEARS (www.gears.vision).

## 3. Computational workflow

### 3.1. Shaders, render state, pipeline, and workflow

In this section, we review computer graphics and GPU programming concepts relevant to the implementation of the GEARS workflow model.

Computer graphics cards are programmed through various specialized Application Programming Interfaces (APIs). Some of them are designed for rendering graphics, while others exploit the parallel architecture of the GPU for general purpose computing. There are two widely used graphics APIs, Direct3D (Hughes et al., 2014) and OpenGL (Shreiner et al., 2013). GEARS employs OpenGL, which is an API based on the C language. Graphics APIs provide an abstraction of the GPU hardware components, implementing a sort of virtual computer, constructed as a pipeline for rendering images.

A pipeline has multiple *stages*, which can be programmable or fixed-function. Fixed-function stages can be customized by setting so-called *render states*. The operation of programmable stages is defined by special pieces of software called *shaders*. Shaders can be written in OpenGL's own shading language, namely GLSL. Pipeline stages perform tasks like filling the GPU memory buffers with arrays of vertices of a shape, applying geometrical operations, breaking down triangles to pixels, and finally, assigning color to the pixels and sending the information to the target graphics buffer.

A drawing sequence that uses a certain configuration of shaders and render states is called a *pass*. The output of a pass is not necessarily a visible image. It can also be an intermediary dataset, which is streamed for further processing to a later pass. Multiple passes can be organized into a chain, which we refer to as a *workflow*.

Modern GPU programming libraries enable low level access to the parallel hardware described above. This allows for efficient compute-intensive rendering, but requires deep understanding of the GPU. GEARS automates the process of programming the GPU to produce stimuli. The method was engineered with the aim to maintain efficient rendering, without demanding advanced knowledge in computer graphics.

### 3.2. Workflow organization in GEARS

**Figure 3:**
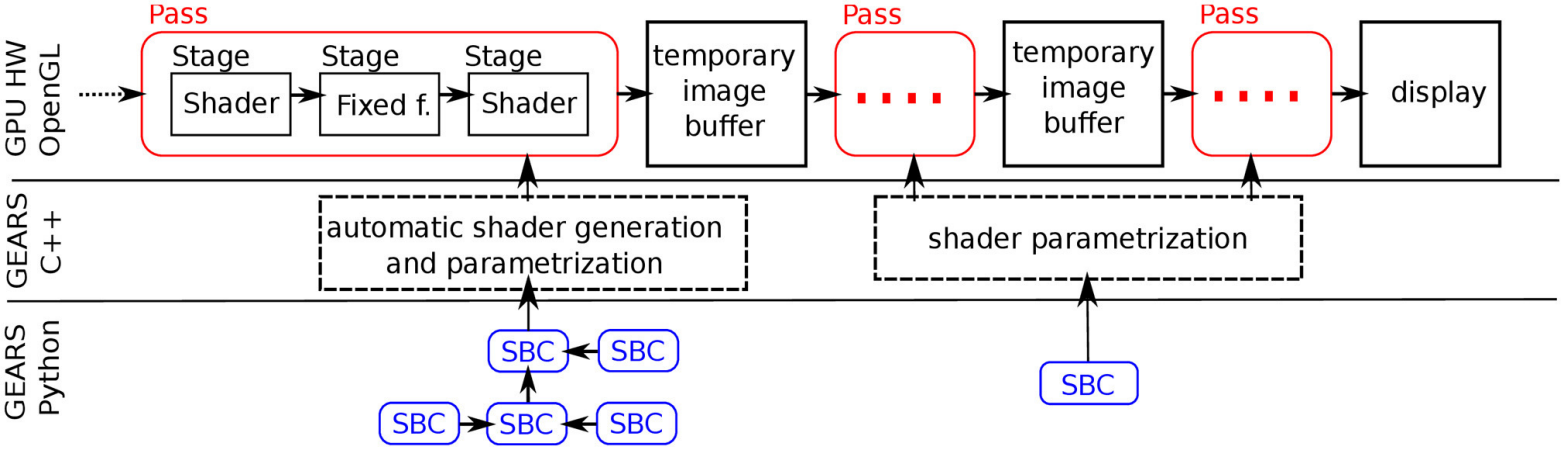
A GPU pass is defined by a full configuration of fixed function stages and programmable shader stages. The GEARS C++ engine automatically generates shaders, parametrizes fixed function and programmable stages, and constructs the workflow of passes. This process is controlled by Stimulus Building Components (SBCs) that define various aspects of stimuli.

In GEARS, all stimuli are constructed as a combination of previously defined *stimulus building components* (SBCs). The scheme is depicted in Figure 3. Our approach is inspired by modern game engine systems, where aspects of game world entities are represented by components that can be freely combined (Eberly, 2006). An SBC is a Python class (program code for creating and managing similar objects) that defines a certain aspect of a stimulus by invoking lower level software methods. Functions of SBCs can be various. Most of them address graphical tasks, either individually (e.g., spatial filtering), or in combination (e.g., providing a shape, pattern, or motion path). Some SBCs individually handle non-graphical features like sound or external devices.

Most widespread software packages designed for a similar purpose use a low number of shaders (GPU programs), while stimuli are implemented by setting appropriate shader parameters. This structure cannot guarantee the flexibility and efficiency that shaders tailored for specific tasks would provide. Moreover, the continuous demand for new stimuli will inevitably exceed the limitations of previously written shaders, while new ones cannot be implemented without resorting to GPU programming.

In GEARS, the core software mechanism dynamically generates graphical (GLSL) shaders from SBC combinations. Therefore, creating custom-made shaders for specific tasks does not need GPU programming expertise. This modular structure is the key to the flexibility and simplicity, and at the same time the high computational power of GEARS. A large library of parametrizable SBCs is provided, which covers the needs for displaying the overwhelming majority of the stimuli we identified in the literature up to 2017 (see Supplementary section 3). Only fundamentally new spatio-temporal light patterning could require extending the capabilities of GEARS, for which several options are discussed in section 3.4.

**Figure 4:**
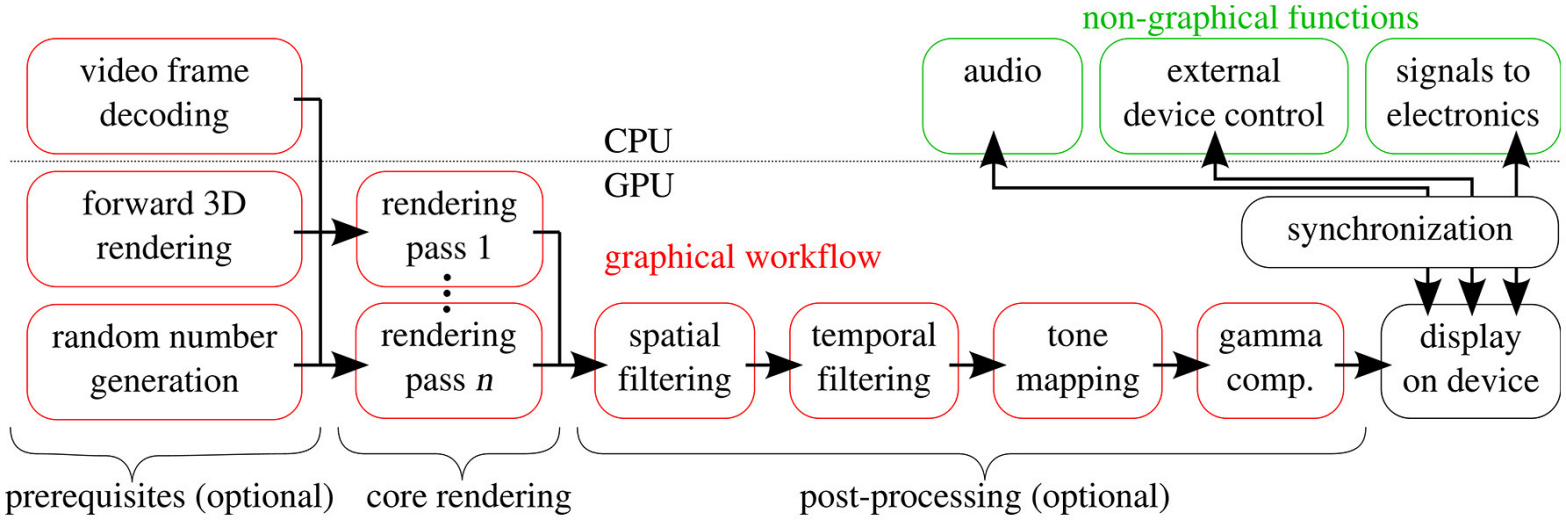
Generation procedure of a single stimulus frame. Prerequisites, core rendering and post processing phases are each composed of passes marked by red boxes.

Figure 4 shows the workflow for rendering (image synthesis) of a single frame of a stimulus. We call the nucleus of the workflow *core rendering*, which is a set of passes designed to produce an intermediate version of a stimulus image, which may be further processed before being displayed on the screen. The core rendering may be preceded by operations like video decoding, random number generation, and/or 3D OpenGL rendering, and can be followed by post-processing steps like spatial and temporal filtering, or gamma compensation.

Non-graphical functions of the stimulus are realized outside of the GPU. The most important of these are emitting signals to drive or to be recorded by the measurement electronics, playing sounds, and controlling external devices. Any new device with a runtime library (e.g., a manufacturer-provided DLL offering functions for controlling the hardware) can be operated from Python scripts using the *ctypes* foreign function library to access the DLL. These tasks, performed by the CPU, are precisely synchronized with the presentation of the images.

#### 3.2.1. Rendering in GEARS

The classical *forward rendering* process draws polygonal shapes defined by vertex positions. This is the rendering model of classic OpenGL, which is straightforward and efficient, but only supports solid polygonal shapes, and limited modes of image composition (alpha blending for transparency, and depth testing for occlusions). These restrictions can be circumvented if the polygons are textured with images. However, using static textures precludes interactivity and limits animation. In order to overcome this problem, textures must be generated dynamically, meaning that their pixels are computed procedurally, using a formula or an algorithm.

In GEARS, forward rendering is only used for 3D content and free-form shapes that are easier to describe using polygons than with membership functions. All the other features are rendered in a per-pixel manner, extending the concept of dynamic texture generation to the entire screen. This approach allows an easy implementation of set operations (e.g., difference or intersection) on multiple shapes, mixing and masking of patterns by linear interpolation, or non-linear spatial distortions with pixel precision. Moreover, soft-edged shapes can straightforwardly be defined, with full control over anti-aliasing to counter artifacts due to finite image resolution. Although this solution might need to process more pixels in case of the simplest, smallest shapes, the overall time cost is still far below the time available for rendering a frame, which is typically more than 1/120 s. We refer to our approach as *pointwise rendering*.

#### 3.2.2. Stimulus building components (SBC)

A large number of SBCs realize specific tasks in the graphical workflow and accompanying non-graphical functions (see Supplementary section 2). Multiple SBCs may influence a single GPU pass in the workflow, and conversely, functionality of a single SBC (notably filterings) may be used by multiple passes (refer back to Figure 3). In this article, we typeset SBC types in bold (e.g., **motion**), and concrete SBC realizations in italics (e.g., *sweep*).

Certain SBC types are responsible for computing prerequisites for image generation. Core rendering passes are composed using combinations of so-called **primer** SBCs that represent elementary stimulus features like patterns or shapes. Combination of **primer** SBCs with each other, as well as with SBCs defining motion, intensity modulation, or distortions, are also allowed. The image resulting from core rendering can be subject to various SBCs performing image processing operations like spatio-temporal filtering or convolution. Additionally, non-graphical tasks performed alongside stimulus rendering are defined by further SBCs. Parameters of all SBCs can be given explicitly or through **interaction** SBCs. These specify bindings for input devices like the mouse or the keyboard.

### 3.3. Details of pivotal functionality

In this section we present the most significant operations, and some respective SBCs, in more detail. An exhaustive description of all SBCs can be found in the user's manual.

#### 3.3.1. Pseudo-random number generation (PRNG)

Several stimuli, like random checkerboards or randomly moving objects, require a large set of (pseudo-)random numbers for each of their frames. Generating random numbers on the CPU, and uploading them on the GPU in every frame might critically slow down the rendering process. Therefore, GEARS incorporates a GPU-based pseudo-random number generator, which produces 2D arrays of random numbers in parallel on the GPU. The SBCs capable of performing this task are called **PRNG** SBCs. One example is the *XorShift128* (Marsaglia, 2003) SBC, which can be parametrized by a random seed. The very same sequence of pseudo-random numbers will arise during the repeated execution of a certain stimulus. Knowing the seed, these sequences can be re-created by the data analysis software, but they can also be exported to text files. Other PRNG SBCs allow shifting the 2D array of random numbers as well. This option is beneficial for generating moving random patterns, where only a small amount of random numbers, covering the newly arising regions, needs to be computed during the rendering of a new frame.

#### 3.3.2. 3D rendering

Implementing a specific virtual reality environment is a demanding task with respect to algorithmic challenges, computational requirements, and content modeling. Most of these are addressed by specialized software called game engines. Our program does not try to implement a full-fledged 3D rendering engine. However, full access to OpenGL functionality is exposed via forward rendering components. Forward-rendered scenes can be used as inputs in combination with any image processing capability.

As a most typical example, GEARS provides flythrough of an easily configurable labyrinth with textured walls. The *labyrinth* SBC, which features parametrizable maze layout and camera path, demonstrates the integration of classical forward rendering using PyOpenGL into the GEARS workflow.

Note that 3D content can also be rendered by *ray casting*, which finds the first intersections between the scene elements and the half-lines from the eye through every pixel. This is a per-pixel method that fits our pointwise rendering scheme. GEARS incorporates a set of further, procedurally generated virtual reality scenes implemented by ray casting (e.g., terrain flyover and 3D fractal exploration).

#### 3.3.3. Core rendering

The strength of core rendering SBCs lies in their easy combinability. We emphasize that combining SBCs is implemented on the level of Python scripting, and the procedure does not assume deep knowledge in GPU programming. For example, moving bars are simply implemented by joining the *sweep* SBC (a **motion** SBC, responsible for moving shapes across the field) to a *rectangle* SBC that defines a rectangle shape. The *mouse wheel* **interaction** SBC adds the possibility of adjusting both the speed and the angle interactively using the mouse.

Numerous SBCs require color parameters. GEARS performs computations in the RGB color space, but is able to convert from widely used color spaces like CMYK, HVS, or XYZ. It is also possible to specify color sensitivity curves or RGB conversion tables for arbitrary receptors. Although the capabilities of the display device may not allow independent stimulation of receptors, GEARS is able to compute and display the stimulation levels resulting from an interactively set color input.

A particularly important SBC is *mixing*, which allows linear interpolation between the pixel values of two **primer** SBCs, taking interpolation parameters from a third **primer**. This allows mixing a background and a foreground pattern according to a certain masking shape. As a result of this operation, the foreground pattern is only visible inside the masking shape, while the background pattern outside of it. Since this foreground/mask/background scheme is sufficient for a majority of the stimuli, GEARS offers a template for the definition of new stimuli that merely requires the listing of the desired masking shape, foreground, and background **primer** SBCs.

Any of these SBCs can be further decorated with **motion**, **modulation**, and **warp** SBCs, all of which have meaningful defaults. For example, a grid of spots with a grating pattern and a black background can be constructed with this template by just listing the *spot* **primer**, *sine* **primer**, and *repeat* **warp** SBCs, respectively.

Generating a smooth interchange (fading) between two patterns is another prominent example of stimulus building: it requires a *mixing* **composition** over two arbitrary **primer** SBCs, with interpolation values from a *fullfield* **primer** with *linear* **modulation**. GEARS also offers a template for this common scenario. For simple cross-fading the only required parameter is the duration, given in seconds or frames.

The *grid points* SBC is a special **warp** component that repeats a **primer** at preset positions, for example on the vertices of a random, rectangular or hexagonal grid. This functionality is especially useful for doing experiments with multielectrode arrays (MEAs), or optogenetically labeled cells. Other SBCs (e.g., the *linear* **motion** component) also accept positions given by custom-defined grid vertex labels, which further simplifies certain tasks performed with MEAs.

*Random checkerboard* SBCs take random numbers generated by the PRNG and display randomly flickering binary, grayscale, or color checkerboards. The shapes used in *polygon mask* SBCs can be interactively edited using the GUI, or they can be loaded from the most common vector graphics file formats. The polygon mask functionality is especially useful if the light stimulus has to be confined to arbitrarily given, free-form regions, or optogenetically labeled cells.

#### 3.3.4. Spatial filtering

GEARS offers a feature which most similar software does not, namely the possibility for real-time spatial and temporal filtering of the rendered frames. Spatial filtering SBCs take the image provided by core rendering, and perform spatial filtering by spatial or frequency domain processing, depending on the choice of the user. Filtering with large kernels is computed more efficiently in the frequency domain, whilst for filterings with small ones, the spatial domain method demands less resources.

Certain filtering operations are nonlinear, and cannot be simply defined by a kernel. GEARS includes SBCs specifying such shaders, e.g., the *median* filtering used for de-noising images, and adding new ones is certainly possible. However, it is more typical to use linear filtering, convolving the image with a kernel given by a mathematical function. New filtering SBCs can easily be specified by modifying the kernel functions of existing ones. For filtering in the Fourier space, the kernel can directly be given in the frequency domain, offering a convenient way to specify low-pass, band-pass, high-pass, or anisotropic filterings.

GEARS offers the possibility to change the kernel parameters in real time, for example by moving the mouse. This operation may incur some transient performance penalty, as the kernels and their transforms have to be recomputed.

#### 3.3.5. Temporal filtering

During temporal filtering the pixel values of the stimulus frames are regarded as *discrete time signals*. Filtering can be performed either by convolution with a temporal one-dimensional filter kernel, or, if the kernel is smooth enough to be well approximated by a composition of complex exponentials, a *linear time invariant* (LTI) signal processing operation is also suitable.

Users may specify the convolution kernel, choosing between direct convolution or using an LTI system. The LTI state representation can be directly given as well. This option is useful, for example, if a theoretical system realization is being investigated, where the differential equations governing the system are known.

SBC realizations that configure temporal filtering include *rectangular, triangular, Hamming*, and *Hann* windows, *exponential* attenuation, and a mixture-of-Gaussians model for the approximation of retina *cell* response. Note that only past frames can be considered in filtering, but this is equivalent to taking future frames into account and presenting the stimulus with a delay.

#### 3.3.6. Histogram manipulation and tone mapping

GEARS has another feature which, besides the possibility of real-time spatio-temporal filtering, is extraordinary among the visual stimulus generating software available for medical and scientific application. This functionality is real-time dynamic tone mapping operations with linear (contrast stretching) and sigmoidal transfer functions, as well as histogram equalization of picture sequences or videos.

These methods are applied to increase the global contrast of an image by adjusting the distribution of image intensities. It is also possible to specify parameters that result in decreasing, and not increasing, the contrast. Tone mapping SBCs include *sigmoid, contrast stretching*, and *equalization*, applying the above functions to map the intensity range into the unit interval. All of these can be set to dynamic operation, adjusting the transfer function for individual frames. The desired contrast and brightness level can be changed interactively, while histogram equalization can be switched on and off during stimulus display.

The intensity values resulting after a filtering operation may go beyond the unit interval. Therefore, a renorming tone mapping operation is necessary. This can be done dynamically for each frame, or using the same mapping for all the frames of a stimulus.

GEARS provides a function for the *in-silico* measurement of the global intensity distribution of the whole stimulus. This extracts and displays the histogram, along with the intensity minimum, maximum, mean, and variance values marked. The user can accept or override the histogram manipulation parameters derived from the measurement. These settings can be changed interactively as well.

#### 3.3.7. Calibrating the gamma curve of the light emitting device

Finally, the stimulus is subject to *gamma compensation*. This is required because most commercial visualizing tools, like projectors, are configured to take human visual psychophysics into account. Accordingly, the same intensity variation produces the same differential change in light intensity perception for both high or low values, according to the Weber-Fechner/Stevens psychophysics law.

However, scientific applications assume a strictly linear relationship between the value specified in the software and the resulting change in light intensity. Therefore, the gamma distortion of the device must be compensated. Since the gamma curve can vary depending on the amount of the time the light emitting device has been used, the safest way is to regularly measure it by an appropriate photometer, and provide the obtained values to GEARS.

#### 3.3.8. Synchronization

Neurophysiology and psychophysics experiments demand exact timing, as well as high precision recording of the onset, offset, and other significant time points of the delivered light stimuli. In order to provide timing data for recording electronics, GEARS emits high precision voltage signals through virtual RS232 ports RTS and BREAK pins (see Supplementary section 4.1, and the Hardware section of the www.gears.vision website). In current computers (2017), RS232 ports are realized by converters plugged into USB ports. Each USB port supports two output channels, and an arbitrary number of them can be handled by GEARS. The user can freely define the signals emitted on these channels. By default, one channel is reserved for signaling the onset and offset of individual stimulus elements, while another one delivers the stop signal arresting the experiment.

Display devices refresh their screens continuously, row by row, pixel by pixel, with a short interval between the refreshes, called the *vertical blank*. The vertical blank occurs at regular intervals (e.g., at 60 Hz). The GPU drives the display from a piece of memory called the *front buffer*, and renders the next frame in a separate *back buffer*. Only after rendering a frame can a *buffer swap* take place, starting to drive the display with the new image data. If the buffer swap occurs during a refresh, the boundary between the refreshed and non-refreshed parts of the screen can show up as a discontinuity. This is called *tearing* (see Supplementary section 4.2). It can be avoided, if the buffer swap happens during the vertical blank. This is ensured by the GPU feature called *vertical sync*, enabled either by user applications of through the manufacturer-provided hardware control application (e.g., NVidia Control Panel). With vertical sync enabled, the GPU does not perform a buffer swap until it receives a VSYNC signal from the display device, indicating a vertical blank. Delaying the buffer swap means the back buffer does not become available for rendering. The GPU blocks requests coming from the CPU to present new frames, thus making the program wait for the buffer swap. This, in case of visual stimulation software, is a useful feature, as it synchronizes the operation of the display device, the GPU, and the CPU.

In a visual stimulation software, tearing is not acceptable, so vertical sync must be enabled. However, it is equally important to display the exact frames required by the experiment, meaning exactly one buffer swap has to be performed at every vertical blank. If the rendering of a frame takes more time than the refresh interval, then the buffer swap cannot occur at a vertical blank, and we have missed an opportunity to display one of the stimulus frames. This is called a *frame drop* (see Supplementary section 4.3).

If tearing is possible, GEARS refuses to execute stimulus sequences and instructs users to adjust hardware settings. The program offers the means to test experiment hardware for frame drops in concrete stimulus sequences. If frames are dropped, a visual or audio warning signal is sent out, and the indices of dropped frames are reported. In this case, either the frame rate, or other performance-critical parameters like FFT resolution need to be adjusted until no frame drops occur. Benchmarks for representative stimuli using an increasing number of hardware configurations are posted to the Hardware section at www.gears.vision continuously. As an alternative to scaling back performance-critical parameters, the real-time condition has to be canceled, and processing of the stimulus has to be performed in advance. Consideration must be given to eventual artifacts resulting from video compression.

Sound signals can be desired not only for research, but also for experiment conducting purposes. GEARS does not want to compete with sound synthesis software, but offers playing sound files. The playback of audio frames is precisely synchronized to graphics display, and to video frames in particular.

### 3.4. Extending GEARS

Since graphical SBCs rely on functions written in the GLSL language, implementing new SBCs is possible but demands more advanced programming skills. For example, creating a new temporal modulation SBC that performs absolute-value-of-cosine modulation can be achieved by slightly modifying the formula (given in GLSL code) in a copy of the SBC script implementing cosine modulation. However, replacing the Fourier transformation used for filtering stimuli with alternative processing (e.g., Zernike transformation) would require editing the C++ source code of the rendering backend layer. Once the new functionality has been added in C++, it is naturally possible to incorporate it into the SBC system, allowing other, non-programmer users to use it through the high-level visually aided scripting interface.

**Forward rendering** SBCs allow access to the entire low-level OpenGL API through PyOpenGL. Resulting images can still be used in the GEARS workflow. It is also possible to create entirely new stimulus classes in Python that do not use the SBC components, but directly interact with the C++ layer to construct shaders and configure other settings. These stimuli can register callback functions to perform tasks in every frame or on other events like keypresses. Thus, while the main focus is on enabling non-programmer scientists to create stimuli easily but efficiently, functionality can be accessed on all of the several lower levels to allow extending GEARS.

## 4. Discussion

### 4.1. Practical aspects and examples of light stimuli

In order to comprehend the rationale behind the stimuli generated by GEARS, in Supplementary section 1 we shortly review the fields of science where these stimuli are applied. For further details on hardware, see Supplementary section 4.

#### 4.1.1. Shapes

Supplementary section 3.1 reviews applications for stimuli with simple shapes like the fullfield, spots, squares, or freeform shapes which can vary in size, shown individually or in groups.

**Figure 5:**
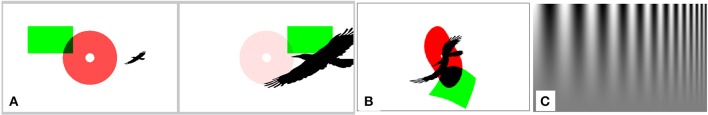
Simple shapes and patterns in GEARS. **(A)** Two frames from a stimulus displaying shapes with changing size and intensity. **(B)** Shapes under non-linear pinch-twist distortion. **(C)** Campbell-Robertson pattern with frequency and contrast gradient.

In GEARS, simple shapes like discs, annuli, or rectangles—and even tilings built of them—can be defined by parametrizing appropriate primer SBCs. New geometrical shapes can easily be constructed by providing their implicit equations or polygon vertices. Free-form shapes can be edited as piecewise Bezier curves (Boehm and Müller, 1999) using an integrated graphics editor. Defining soft shapes with fuzzy membership functions are also possible. SBC parameters allow the setting of the luminance, color, and size of the shapes. Switching them on or off with high temporal precision, or altering their size and position is supported. Figure 5 shows some examples rendered in GEARS.

#### 4.1.2. Motion

Supplementary section 3.2 reviews applications for stimuli with shapes, like spots, bars, and freeform shapes, moving at various velocities and orientations.

**Figure 6:**
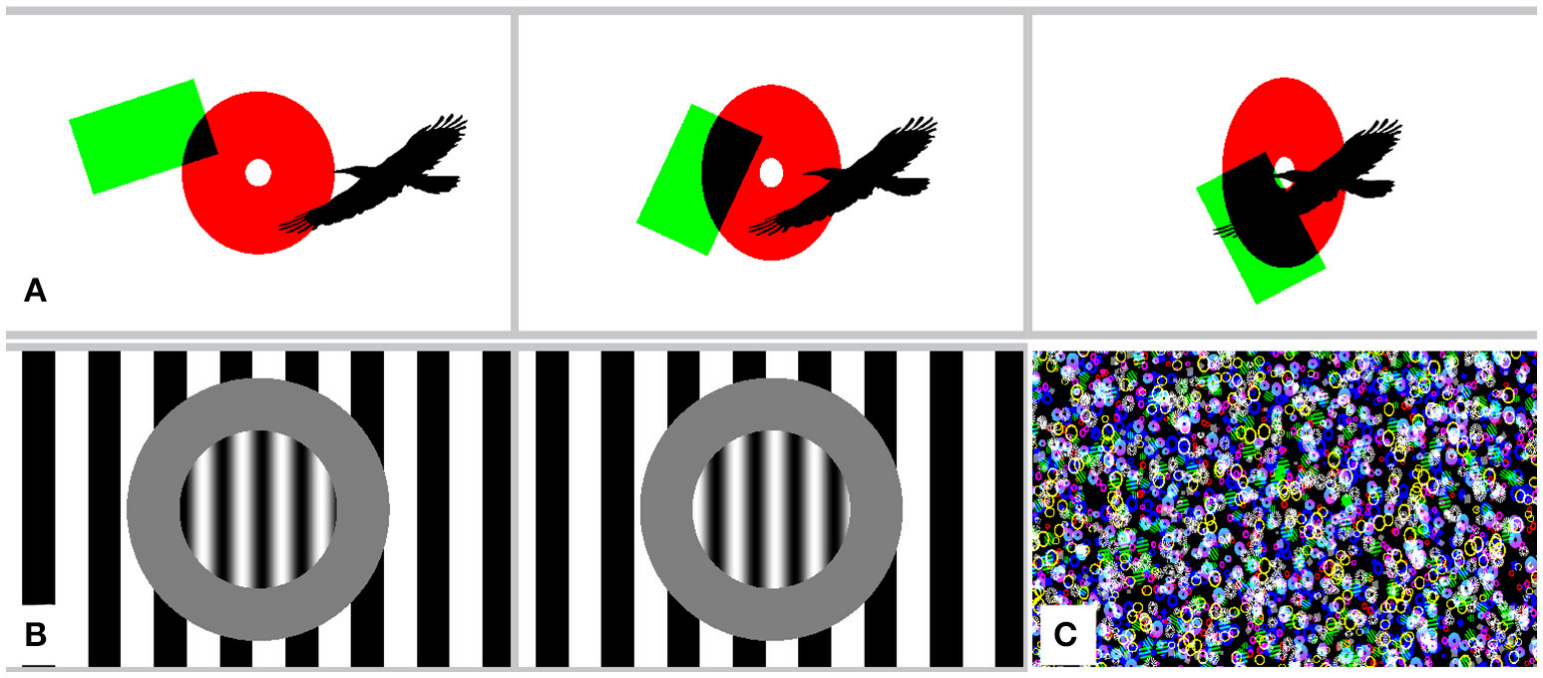
Sample frames taken from moving (shifting and rotating) patterns: **(A)** Moving and deforming shapes, **(B)** Square grating and sinusoid grating, separated by an annulus, and moving in opposite directions. Note that implementing phase inversion is also straightforward. **(C)** 5,000 shapes with random size, orientation, and motion, rendered without performance issues.

GEARS offers a broad variety of **motion** SBCs that can be attached to arbitrary **primer** SBCs (see Figure 6 for examples). In the *linear* **motion** SBC, either the velocity, or the time necessary for a shape to cross the screen has to be specified. The *free-form* **motion** SBC allows arbitrary motion paths, interpolated from provided control points of the trajectory and the velocities of the shapes at these points. Superimposing random shaking over any smooth motion is also possible. Appropriate SBCs allow the speed and direction of motion to be interactively changed by pressing certain keys or by moving the mouse during the experiment.

#### 4.1.3. Temporal modulation

Supplementary section 3.3 reviews applications for stimuli with shapes with contrast or intensity regularly modulated in space (grids) or in time (sinusoidal or other waveform with fixed or varying period).

**Figure 7:**
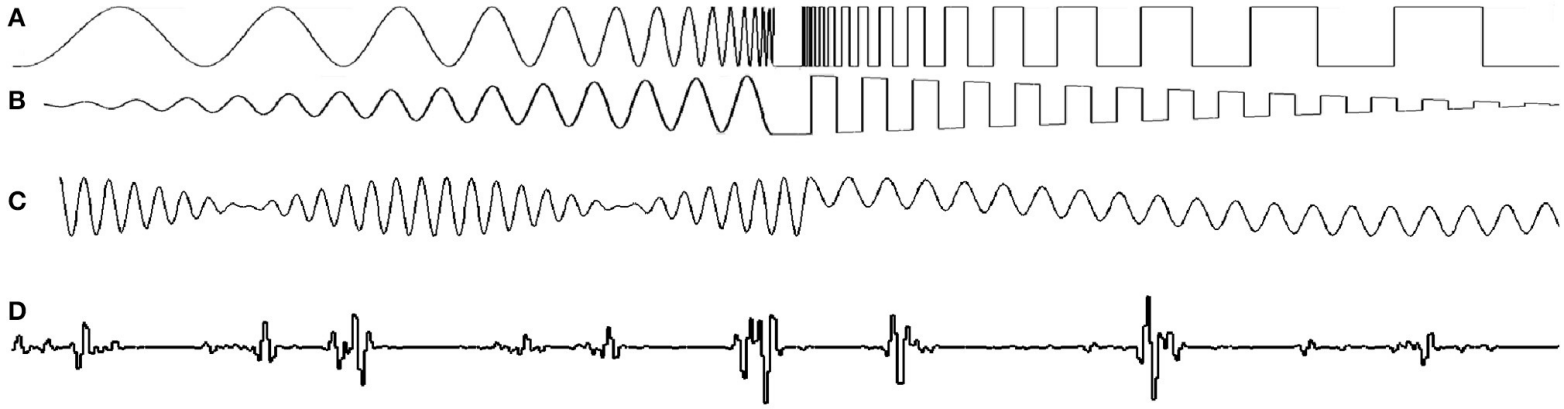
Intensity plotted vs. time for various modulation options: **(A)** changing frequency on sine and square waves, **(B)** changing amplitude on sine and square waves, **(C)** combinations of dissimilar and similar frequency components, and **(D)** synthesis from a multitude of frequency components.

GEARS allows the realization of stimuli of this type by combining **modulation** and **primer** SBCs. Ready-made SBCs for intensity modulation include *linear* fading, *sinusoidal* and *square* waveforms (Figure 7 shows some examples). Note that frequency and amplitude can be configured to change during the stimulus, by giving their initial and final values. New **modulation** SBCs can be realized by providing the intensity value as a function of time.

#### 4.1.4. Flickering checkerboards

System identification is an efficient tool that can promote the understanding how certain subsystems of the visual tract work, provided that the input (the light stimulus) and the output (voltage trace of certain neurons) are known. In visual neuroscience, typically nonparametric system identification is applied. The visual input is spatio-temporal, and usually white noise implemented by a flickering checkerboard is used for probing (Chichilnisky, 2001). Supplementary section 3.4 reviews applications for stimuli using pseudo-random flickering checkerboards.

**Figure 8:**
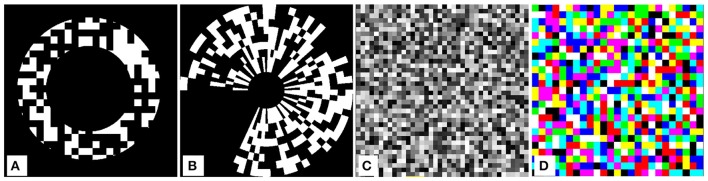
Random checkerboard stimuli. Binary (black and white) figures are colored depending on whether the magnitude of the random numbers, spread within [0…1], is greater or less than 0.5. Gray shades are generated by linear mapping of random numbers to the dynamic range. Colored checkerboards are generated using three sets of random numbers, for the red, green and blue channels. **(A)** Binary, restricted to annulus. **(B)** Binary, in polar coordinates. **(C)** Grayscale. **(D)** Color.

The random numbers, generated by the *XorShift128* SBC for each frame, are accessible for other components in the stimulus as well, and they can be exported to files for the evaluation of measurement results. Variants, like shifting the array while introducing randoms at the border, are also supported. **Image** SBCs that make use of the random numbers to render a checkerboard can be defined (Figure 8), including binary random and Gaussian white noise checkerboards rendered in grayscale and in color, with adjustable luminance and contrast levels. Note that these randoms can be used for other purposes, like randomly moving a high number of shapes.

Supplementary section 3.5 reviews applications creating virtual environments for stimuli using pseudo-random flickering checkerboards.

#### 4.1.5. Natural videos and virtual environments

**Figure 9:**
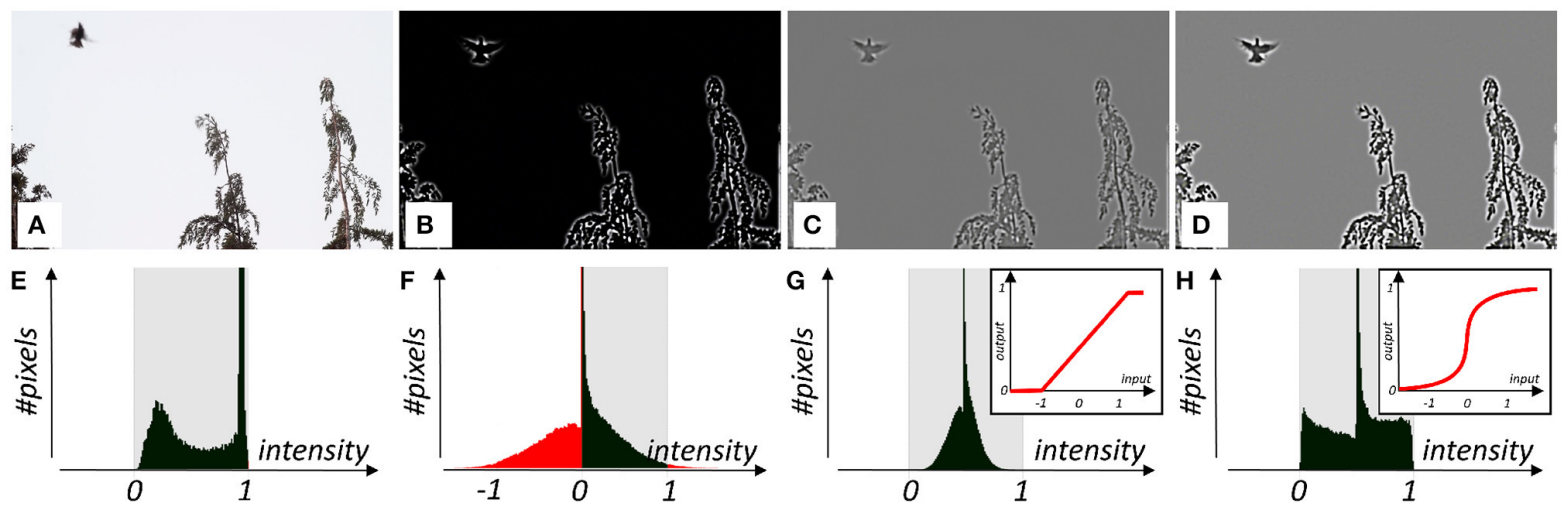
Real-time filtering and subsequent tone mapping on a frame of a natural video: **(A)** unfiltered frame, **(B)** difference-of-Gaussians filtering applied, **(C)** subsequent tone mapping by linear transfer function, **(D)** subsequent tone mapping by sigmoidal transfer function. **(E–H)** Subfigures in the second row show respective histograms measured and displayed in GEARS. Histogram segments extending beyond the dynamic range [0…1] are shown in red. These regions are “squeezed back” by the tone mapping transfer function to the dynamic range. Insets in the last two figures show the transfer functions used.

Beside simple geometric shapes, examination of the visual tract also requires more complex, realistic patterns (Felsen and Dan, 2005; Hyvärinen et al., 2009), which are usually provided as a video record. GEARS can stream videos of mainstream formats (avi, asf, mov, and mp4) into GPU textures, and all per-frame real-time processing options available in our software can be applied. Most importantly, videos can be subjected to real-time and interactive spatio-temporal filtering, contrast manipulation, and brightness adjustment. The real-time histogram equalization can be switched on and off interactively. Ready-to-use SBCs include various filtering options like edge detection and contrast enhancement (Figure 9). Local image distortions can also be performed by applying **warp** SBCs.

In order to extract statistical properties of videos, GEARS features global or framewise histogram generation, as well as Fourier transform, average Michelson and RMS contrast determination, and entropy calculation of individual frames.

Computationally-generated stimuli can be projected via specialized imaging systems, on hemisphere and torus-shaped display screens covering most of the visual field (Takalo et al., 2012; Geuss et al., 2015). Depending on the mirrors and the screen used, different predistortion (warping) can be required in order to lead to a realistic final image (Bourke, 2004, 2005). GEARS offers **warp** SBCs for custom transformation which can be parametrized for projection onto spherical surfaces via flat or spherical mirrors (see Supplementary Figure S10). Supplementary section 3.5 reviews applications of virtual reality, and simulation of various environments like labyrinths.

Virtual Realities may be realized by **forward rendering** SBCs. For example, in case of the *labyrinth*, important features of the journey, like camera path and labyrinth layout can simply be parametrized. Camera motion can also be controlled by feedback from an external device (e.g., a treadmill). Texturing walls with patterns like stripes or scale-free images is also possible. For rendering 3D environments by ray casting of procedural scenes (like fractal-like terrain, vegetation, industrial, and indoor scenes), shaders can be found in the public domain in great abundance (Quilez, 2014) (see Supplementary Figure S11).

Handling multiple displays is also allowed by extending the desktop to multiple screens and setting the field size to their combined extents. **Composition** SBCs to assign different stimuli to desired parts of the field are provided. Prerequisites are shared by stimuli appearing on different monitors, and filtering is applied to them uniformly.

#### 4.1.6. Stimuli for visual psychophysics

Classical psychophysical methods like various types of adjustments and judgments all require the recording and statistical analysis of the response of the subject (Pelli and Farell, 1995; Lu and Dosher, 2013). GEARS takes feedback by mouse and keyboard, and other options including specialized devices or microcontroller can also be implemented. All feedback events are logged. Static or dynamic white noise can be added, for example, by combining any stimulus with a specially adjusted *checkerboard* SBC set to the same dimensions as the screen resolution.

Some of the classic examples of visual stimuli and illusions are already implemented in GEARS (e.g., drifting Gabor patches, rotating snakes (see Supplementary Figure S12), or Hermann grids). An effective tool in investigating visual illusions is the parametrization of the images between a form where the effect appears, and another one where it is not present (Schrauf et al., 1997). In order to address this task, GEARS can use image files as **primer** SBCs, and fade between them. The SBC toolkit of GEARS allows easy implementation of further visual illusion or psychophysics stimuli.

#### 4.1.7. Interactive, real-time filtering, and histogram equalization

Real-time image processing plays an important role in the fields where fast feature extraction from, or quality enhancement of a video stream is necessary. Pre-processing for visual prostheses, quality enhancement of the images delivered by night vision cameras (Jungenfelt and Raski, 2012) or other recorders working in reduced visibility (Beckman, 2015) all require various image manipulation algorithms. Real-time operation requires substantial computing power. A series of commercially available video processing systems (Beckman, 2015) apply special hardware, while some of them (Ikena ISR, 2016) perform the task using commercial software. Edge enhancement (Kwon et al., 2012) and histogram equalization (Livingston et al., 2011) are the most important algorithms in the applications mentioned above.

**Figure 10:**
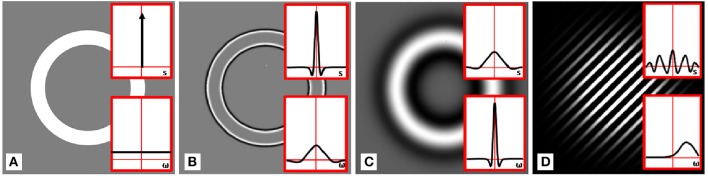
Real-time filtering on an annulus. Insets show 1D cross-sections of the spatial and frequency domain plots of filter kernels, having zero integral. **(A)** Unfiltered frame, **(B)** after small-sized difference-of-Gaussians filtering, **(C)** after larger difference-of-Gaussians filtering. Note that after a double-Gaussian filtering with zero integral, a homogeneous area is mapped to zero (black), while around the edges, intensity values beyond the [0…1] dynamic range can occur. In order to fit into the dynamic range, a linear mapping from the resulting intensity interval into the dynamic range has been applied. Therefore, the regions with zero intensity get a value corresponding to a gray shade. **(D)** Small spot filtered with phase-shifted Gaussian (Gabor) filter.

GEARS is able to perform such operations, including like real-time image enhancement, using the computing power of the GPU. In the figure below (Figure 10), we present as example edge enhancement realized by a difference-of-Gaussians filtering, which is followed by two possible tone mapping operations.

### 4.2. Validation and application

We validated stimulus timing using submillisecond-precision electronic light sensors. We found that stimulus durations were in absolute agreement with the prescribed values, accounting for the time quantization due to the finite refresh interval of the display device. We measured a constant, invariant time difference between the display of a stimulus frame and the recording of a simultaneously emitted signal in the measurement electronics. Details can be found in Supplementary section 4.1.

We validated geometric accuracy by measuring object dimensions both on direct screenshots and on images projected onto the specimen. This was accomplished by analyzing the images of a CCD camera coupled to the microscope that was installed for the purposes of the experiments on rodent retina.

The software was used for various experiments performed on C57/BL6 mouse retina on USB-MEA256 multielectrode array manufactured by Multichannel Systems. The sample preparation and the technical details of the recordings were conducted as described in Cronin et al. (2014). Details and results of the experiments are given in the Supplementary section 6, we only summarize them here.

We reconstructed linear kernels of retinal ganglion cells and their upstream circuitries. This was performed by projecting checkerboards implementing different forms of white noise onto the retina.

A stimulus sequence with segments where the illumination uniformly decreases has been elaborated to investigate the response of a subset of OFF-type retinal ganglion cells in mice. This was used to measure the dependence of cell behavior on the rate of light decrease.

We investigated changes in spiking characteristics of the retina when videos with sudden intensity histogram changes were displayed. We measured activity patterns suitable for ganglion cell classification.

Experiments described in Supplementary sections 6.1 and 6.2 were performed according to standard ethical guidelines (European Communities Guidelines on the Care and Use of Laboratory Animals, 86/609/EEC) and were approved by the Veterinary Department of the Canton of Basel-Stadt. Experiments were performed on 4–8 week old female C57Bl/6 mice that were maintained on a 12 h light/dark cycle.

Experimental procedures described in Supplementary section 6.3 were carried out in compliance with the institutional guidelines at the NMI. Housing and euthanasia of animals were compliant with applicable German and European law for the protection of animals used for scientific purposes and were approved by the Regierungspräsidium Tübingen, Germany (AZ 35/9185 82-2).

## 5. Conclusions

We have implemented a universal, flexible, and user-friendly light stimulus generating software (GEARS, www.gears.vision) for conducting research in visual science, with the ability to perform computationally intensive operations. These operations comprise, among others, real-time spatio-temporal filtering, contrast manipulation, histogram equalization, and image generation demanding a large amount of pseudo-random numbers. Parameters of the stimuli can be varied interactively during the experiments. Shaders drawing the images are generated from a large number of elementary stimulus building components. This technique, often encountered in game programming, offers great flexibility when new stimuli are assembled. Since GEARS offers an intuitive visually guided script editor, editing and configuring new stimuli does not require deep knowledge in GPU programming. The software meets the widest spectrum of requirements identified in existing applications in the electrophysiology of the visual tract, psychophysics, and ophthalmology.

The unique functions of GEARS will help physiologists, ophthalmologists and psychophysicists with a demand for computationally intensive tasks. We expect that a broad and constructive user community will rapidly build up.

## Author contributions

LS was responsible for low-level system design and architecture, did the majority of high-level programming and contributed to the writing of the paper. ÁK contributed to the development of numerous low-level algorithmic features, performed data analysis and prepared the figures. GZ worked in testing the software and doing MEA experiments with rodent retina. PH initiated and coordinated the project, determined the content of GEARS, tested the software, performed MEA experiments with rodent retina and contributed to the high-level stimulus scripting and to writing of the paper.

### Conflict of interest statement

The authors declare that the research was conducted in the absence of any commercial or financial relationships that could be construed as a potential conflict of interest.

## Supplementary material

The software and the user's manual can be downloaded from www.gears.vision. Additional videos and screenshots showing the capabilities of GEARS can also be found on this website. In the Hardware section we publish documents describing measurement methodology and equipment. In particular, we provide details on the RS232/TTL adapter that is used to connect the computer running GEARS with the measurement electronics.

The supplementary text published with this article gives more background on visual stimuli encountered in visual sciences. It includes additional technical details on GEARS, including information on components types, GUI, and display synchronization. Results of validation experiments are also presented.

## References

[B1] Beckman F. (2015). Want to See What We See? Pushing the Limits of Underwater Video. Lund: LYYN AB.

[B2] Boehm W. Müller A. (1999). On de Casteljau's algorithm. Comp. Aided Geomet. Design 16, 587–605.

[B3] Bourke P. (2004). Dome Projection on a Budget, Also Known as Mirrordome. Available online at: http://paulbourke.net/dome/mirrordome/ (Accessed on Feb 02, 2016).

[B4] Bourke P. (2005). Using a spherical mirror for projection into immersive environments, in Proceedings of the 3rd International Conference on Computer Graphics and Interactive Techniques in Australasia and South East Asia, (Dunedin: Graphite (ACM Siggraph)), 281–284.

[B5] Chichilnisky E. (2001). A simple white noise analysis of neuronal light responses. Netw. Comput. Neural Syst. 12, 199–213. 10.1080/713663221 11405422

[B6] Cronin T. Vandenberghe L. H. Hantz P. Juttner J. Reimann A. Kacsó A. E.. (2014). Efficient transduction and optogenetic stimulation of retinal bipolar cells by a synthetic adeno-associated virus capsid and promoter. EMBO Mol. Med. 6, 1175–1190. 10.15252/emmm.201404077 25092770 PMC4197864

[B7] DepthQ 360 DLP Projector (2016). Cambridge Research Systems. Available online at: http://www.crsltd.com/tools-for-vision-science/displays/depthq-360-dlp-projector/

[B8] E-Prime2 (2016). A Suite of Applications Used to Design, Generate, and Run Computerized Behavioral Experiments. Available online at: https://www.pstnet.com/eprime.cfm (Accessed on Feb 02, 2016).

[B9] Eberly D. H. (2006). 3D Game Engine Design: A Practical Approach to Real-Time Computer Graphics. Bosa Roca: CRC Press.

[B10] Felsen G. Dan Y. (2005). A natural approach to studying vision. Nat. Neurosci. 8, 1643–1646. 10.1038/nn1608 16306891

[B11] Geuss M. N. Stefanucci J. K. Creem-Regehr S. H. Thompson W. B. Mohler B. J. (2015). Effect of display technology on perceived scale of space. Hum. Fact. 57, 1235–1247. 10.1177/0018720815590300 26060237

[B12] Hodgson N. (2016). Scintilla. Available online at: https://riverbankcomputing.com/software/qscintilla/intro

[B13] Hughes J. F. Dam A. V. Foley J. D. Feiner S. K. (2014). Computer Graphics: Principles and Practice. Boston, MA: Pearson Education.

[B14] Hyvärinen A. Hurri J. Hoyer P. O. (2009). Natural Image Statistics: A Probabilistic Approach to Early Computational Vision., Vol. 39 London: Springer Science & Business Media.

[B15] Ikena ISR (2016). Real-Time computer Vision and Video Enhancement. Burlingame, CA: MotionDSP Inc.

[B16] Jungenfelt N. Raski T. (2012). Contrast Enhancement, Denoising and Fusion in Dark Video for Applications in Automobile Safety. Master Thesis, Chalmers University of Technology.

[B17] Klette R. (2014). Concise Computer Vision. London: Springer.

[B18] Kubilius J. (2014). A framework for streamlining research workflow in neuroscience and psychology. Front. Neuroinformat. 7:52. 10.3389/fninf.2013.00052 24478691 PMC3894454

[B19] Kwon M. Ramachandra C. Satgunam P. Mel B. W. Peli E. Tjan B. S. (2012). Contour enhancement benefits older adults with simulated central field loss. Optom. Vis. Sci. 89:1374. 10.1097/OPX.0b013e3182678e52 22885784 PMC3438282

[B20] Livingston M. A. Garrett C. R. Ai Z. (2011). Image Processing for Human Understanding in Low-visibility. Washington, DC: Technical Report, DTIC Document.

[B21] Lu Z.-L. Dosher B. (2013). Visual Psychophysics: From Laboratory to Theory. London: MIT Press.

[B22] Marsaglia G. (2003). Xorshift rngs. J. Statist. Soft. 8, 1–6. 10.18637/jss.v008.i14

[B23] Mathôt S. Schreij D. Theeuwes J. (2012). OpenSesame: an open-source, graphical experiment builder for the social sciences. Behav. Res. Methods 44, 314–324. 10.3758/s13428-011-0168-7 22083660 PMC3356517

[B24] Muller E. Bednar J. A. Diesmann M. Gewaltig M.-O. Hines M. Davison A. P. (2015). Python in neuroscience. Front. Neuroinform. 9:11. 10.3389/fninf.2015.00011 25926788 PMC4396193

[B25] OpenSesame (2016). An Open-Source, Graphical Experiment Builder for The Social Sciences. Available online at: http://osdoc.cogsci.nl/ (Accessed Feb 02, 2016).

[B26] Peirce J. W. (2009). Generating stimuli for neuroscience using PsychoPy. Front. Neuroinform. 2:10. 10.3389/neuro.11.010.2008 19198666 PMC2636899

[B27] Pelli D. G. Farell B. (1995). Psychophysical methods, in Handbook of Optics, Vol. 1, eds Bass M. Van Stryland E. W. Williams D. R. Wolfe W. L. (New York, NY: McGraw-Hill), 29.21–29.13.

[B28] Presentation (2016). A Stimulus Delivery and Experiment Control Program for Neuroscience. Available online at: https://www.neurobs.com/ (Accessed on: Feb 02, 2016)

[B29] Psychopy (2015). Psychology Software in Python. Available online at: http://www.psychopy.org/ (Accessed on: Feb 02, 2016).

[B30] Psychtoolbox (2016). Psychophysics Toolbox Version 3. Available online at: http://psychtoolbox.org/ (Accessed on: Feb 25, 2016).

[B31] Quilez I. (2014). Canyon. Available online at: https://www.shadertoy.com/view/MdBGzG (Accessed on Feb 02, 2016).

[B32] Schrauf M. Lingelbach B. Wist E. R. (1997). The scintillating grid illusion. Vis. Res. 37, 1033–1038. 9196721 10.1016/s0042-6989(96)00255-6

[B33] Shreiner D. Sellers G. Kessenich J. Licea-Kane B. (2013). OpenGL Programming Guide: The Official Guide to Learning OpenGL, version 4.3. Ann Arbor, MI Addison-Wesley.

[B34] Software Comparision (2016). Comparison of Behavioral Experiment Software. Available online at: https://en.wikipedia.org/wiki/Comparison_of_behavioral_experiment_software (Accessed on: Feb 25, 2016).

[B35] Spapé M. Verdonschot R. Dantzig S. V. Steenbergen H. V. (2014). The E-Primer: An Introduction to Creating Psychological Experiments in E-Prime®. Leiden: Leiden Univerisity Press.

[B36] Strasburger (2015). Software for Visual Psychophysics. Available online at: http://www.hans.strasburger.de/psy_soft.html (Accessed on: Feb 25, 2016).

[B37] Straw A. D. (2008). Vision Egg: an open-source library for realtime visual stimulus generation. Front. Neuroinformat. 2:4. 10.3389/neuro.11.004.2008 19050754 PMC2584775

[B38] Takalo J. Piironen A. Honkanen A. Lempeä M. Aikio M. Tuukkanen T.. (2012). A fast and flexible panoramic virtual reality system for behavioural and electrophysiological experiments. Sci. Rep. 2:324. 10.1038/srep00324 22442752 PMC3310229

[B39] VisionEgg (2009). An Open-Source Library for Realtime Visual Stimulus Generation. Available online at: http://visionegg.org/ (Accessed on: Feb 25, 2016).

[B40] Yoonessi A. Yoonessi A. (2011). A glance at psychophysics software programs. Basic Clin. Neurosci. 2, 73–75.

