# Supplementary material for: Interactive Light Stimulus Generation with High Performance Real-Time Image Processing and Simple Scripting
Source: Front Neuroinform. 2017 Dec 13;11:70. doi: 10.3389/fninf.2017.00070 (PMC5733361; doi:10.3389/fninf.2017.00070)
Supplement: Supplementary file 1 [file Table1.pdf]

Supplementary Material: Interactive Light Stimulus Generation with High Performance Real-time Image Processing and Simple Scripting

László Szécsi, Ágota Kacsó, Günther Zeck and Péter Hantz
*Correspondence:
László Szécsi:
szecsi@iit.bme.hu

1 SHORT REVIEW OF THE VISUAL TRACT

The vertebrate retina is a light-sensitive layered tissue, which can be considered an extension of the central nervous system (Demb and Singer, 2015; Snowden et al., 2012). Phototransduction for image-forming vision occurs in rod and cone photoreceptor cells. The visual information, already pre-processed by the retina, is transmitted to the brain by spiking retinal ganglion cells. System identification techniques revealed that most retinal circuitries upstream of these cells, in some approximation, can be regarded as a linear system followed by a nonlinear Poisson spike generator (Chichilnisky, 2001). Ganglion cells in the vertebrate retina can be divided in three main classes. *On* cells increase spiking to the onset of light, while *off* cells to the cessation of light, and *on-off* cells to any change in illumination. They collect signals from a receptive field consisting of concentrically arranged ON and OFF regions. The central part, which determines the on/off/on-off character of the cell, is encompassed by an antagonistic surround. This structure can be modeled by the difference of two Gaussian surfaces and the resulting *Mexican hat* sensitivity profile implements a spatial filtering, namely a natural edge detector (Rodieck, 1965). Temporal filtering is shown to play a role in dynamic adaptation of photoreceptors (Clark et al., 2013) (Smirnakis et al., 1997). Specialized retinal ganglion cell types preferentially respond to, among others, orientation of elongated shapes, objects moving in certain directions, approaching shapes, onset or offset of illumination as well as to different wavelength (Wässle, 2004) (Joselevitch and Kamermans, 2009). Information is transmitted to the brain via parallel channels (Wässle, 2004; Dowling, 1987).

The optic nerve, composed by the axons of the ganglion cells, transmits the image-forming and the non image-forming signal (Troncoso et al., 2011) to the latero-geniculate nucleus (LGN). The image-forming signals are further transmitted, by different neurons, to the primary visual cortex. Receptive fields of the neurons in the LGN have a similar center-surround configuration as retinal ganglion cells, while those of the cortical cells can be more complex. In LGN and in the primary visual cortex, namely V1, the neurons are organized in a so-called retinotopic mapping, that is, they are located along a transformed 2D representation of the visual image formed on the retina (Baker, 2013). Direction and orientation selective cells are also present in the primary visual cortex. Receptive field weight functions of so-called simple

cells in V1 area of mammalian visual cortex can satisfactorily be modeled by Gabor functions (Daugman, 1980)).

Visual psychophysics investigates relationships between sensations and the underlying light stimulus. A set of experimental arrangements target the determination of the psychometric function, that is, the connection between a given feature of the stimulus (like brightness or velocity) and the observer's response (Lu and Dosher, 2013). In stimulus adjustment tasks, the subject is actively controlling the level of the stimulus in order to match a certain criterion (Pelli and Farell, 1995; Lawless, 2013). In further experimental arrangements, the subjects are asked to take a decision, or make an adjustment. During *yes/no* decision tasks the observer is presented with one stimulus, and is asked to answer a polar question, while for *forced choice* experiments a number of stimuli are presented, and the subject has to decide between them. *Visual illusions* occur when the perceived images differ from objective reality (Michaelbach, 2016). *Physiological illusions* are caused by physical characteristics of the retina or the image processing centers of the brain, while *cognitive illusions* occur when a figure is ambiguously or incorrectly interpreted due to some prior knowledge or assumptions. *Pathological illusions* emerge due to some malfunction in the visual tract.

Note that the development of image enhancement can be inspired by the image processing taking place in the visual tract of different organisms (Herault et al., 2015) (Warrant et al., 2014) (Benoit et al., 2010) (Medathati et al., 2016).

2 ENUMERATION OF STIMULUS BUILDING COMPONENTS

We may organize types of stimulus building components (SBCs) according to the phase they play a role in: prerequisite computation, core rendering, post-processing, and non-graphical tasks.

SBC types for computing prerequisites for image generation are **pseudo-random number generator** SBCs, which produce random numbers en-masse, profiting from the parallel architecture of the GPU, **particle system** SBCs for many-item simulations, and **forward rendering** SBCs, responsible for 3D-rendered content. GEARS uploads images and video frames (decoded by FFMPEG (Bellard et al., 2016)) to the GPU automatically, according to the configuration of later passes.

Core drawing passes are composed of **primer** SBCs, which set up the pointwise rendering of images, giving the pixel color as a function of 2D position and time. This is the most populous group of SBCs, with a wide range of functionality. Some of these SBCs generate patterns or shapes (*e.g.* a sine grid or a disc) procedurally, while being extensively parametrizable. Custom-made new components can easily be implemented by providing the desired formulae defining the pattern. Further **primer** SBCs display video frames, or images either loaded from files, or 3D content from **forward rendering**. Another subset of SBCs use random numbers for producing band-limited or white noise, or randomly positioned and animated shapes.

Members of the **image composition** SBC group can be used to combine multiple **primer** SBCs (e.g. just two spots side-by-side). They are implemented by function composition: **primer** functions are substituted into **composition** functions, yielding a new **primer**. Patterns and shapes (both defined by **primers**) are combined this way. Building a full operation tree is possible, using simple addition, subtraction, and multiplication operators in the Python scripts. **Polygon mask** SBCs allow the definition a binary image by providing the control vertices of its contour(s). This is helpful for drawing free-form shapes, where a formulaic definition is not feasible. Such shapes can be used, among others, for masking in the pointwise rendering scheme.

Several types of SBCs modify raw **primer** SBCs: **motion** SBCs change, as a function of time, scale, orientation and position; **modulation** SBCs vary the intensity or contrast, **warp** SBCs can be used to distort renderings, *e.g.* for toroidal or spherical screens, or tile them indefinitely; **time warp** SBCs distort or reverse the time scale, or introduce periodicity. Modified **primer** SBCs accept further modifiers, allowing them to be chained up indefinitely.

The image resulting from core drawing can be subject to various image processing operations. SBC types performing these tasks are the following: **spatial filtering** SBCs compute a convolution with a filter kernel, either in the spatial or the frequency domain; **temporal filtering** SBCs convolve the image stream with a temporal filter kernel, or channel it through a linear data processing system; **gamma compensation** SBCs cancel the nonlinearity introduced by the light projecting hardware.

Additionally, non-graphical tasks performed alongside stimulus rendering are defined by further SBCs. **audio** SBCs play various audio file formats, and **signal** SBCs provide synchronization, start and stop signals for the recording electronics.

3 MAINSTREAM LIGHT STIMULI IN THE FIELDS OF APPLICATION

3.1 Simple shape(s) with varying size and different contrast

A simple way of investigating the light response of the retina and the structure of receptive fields along the visual path are the stimulations with light and dark spots, squares or annuli, having a series of fixed (Kuffler, 1953)(Farrow et al., 2013), or a continuously altered (Münch et al., 2009) diameter. Their sudden translocation might also be required (Livingstone and Conway, 2003).

Application of optogenetic actuators (proteins that modify the activity of the cell in which they are expressed when that cell is exposed to light) often needs the illumination of custom-defined shapes (Packer et al., 2013). Showing simple shapes with different contrast, color and size is a basic requirement in visual psychophysics as well (Lu and Dosher, 2013).

3.2 Shapes moving along and oriented in different directions

Direction and orientation selective cells of the visual cortex were discovered in the 1950's, as a result of the seminal work of D. Hubel and Th. Wiesel: they extensively applied positive and negative contrast moving bars spreading through the entire field of view. Shorter shapes or even spots have also been applied. This latter stimulus might result in more intense spiking, due to less lateral inhibition (Hubel and Wiesel, 1959). Couple of years later H. Barlow and W.R. Levick revealed that direction selectivity is present in the retina as well (Barlow and Levick, 1965). Investigating the so-called simple and complex cells of the visual cortex require stationary and moving bars or gratings having arbitrary orientation (Hubel et al., 1995).

A great variety of stimuli were used during subsequent research on direction and orientation selective cells. Whole-field drifting gratings with different spatial frequencies were already applied in the '60s for stimulating cat retina (Enroth-Cugell and Robson, 1966). It has also been discovered that certain ganglion cells specifically respond to motion reversal of a dark bar (Schwartz et al., 2007). Stimuli divided into a small central region and a periphery have been used to decipher how local motion is distinguished from the global retinal image drift. The central part is composed of a jittered grating, while the periphery consisted either by a differently jittered grating or a uniform gray scene (Ölveczky et al., 2003). Similar complex stimuli were used to understand wide-field inhibition on direction selectivity processing: while a central grating was drifting in a certain direction, the peripheral one, having a different wavelength and divided

from the central stimulus by a small gray annulus, had a different orientation and drifting speed (Hoggarth et al., 2015). Cells sensitive for moving complex shapes have also been discovered (Zhang et al., 2012). Beside drifting gratings, phase reversing static grating in the center or the periphery of the receptive field of a retinal ganglion cell were used to map the nonlinearities of their receptive field (Demb et al., 1999).

3.3 Intensity oscillation in time

Temporal filtering properties of neural circuitries involving photoreceptor cells can be investigated by presenting them sinusoidally-modulated stimuli with various frequencies. It has been shown that circuitries upstream to the retinal ganglion cells act as bandpass or lowpass filters, with parameters depending on the size and the contrast of the stimulus (Van de Grind et al., 1973)(Shapley and Victor, 1978). Spatially uniform light stimulus composed of several sinusoidally modulated signals (Fourier synthesis), apparently having a randomly changing intensity in time has been applied in deciphering the encoding properties of bipolar cells (Ichinose et al., 2014). Stimuli with sweeping contrast or frequency have been used in classification of retinal ganglion cells (Baden et al., 2016)(Sakai et al., 1988). A linearizing effect of an auxiliary signal superimposed to a sinusoidal one in goldfish retina has been reported (Shapley, 2009).

3.4 Flickering checkerboards

System identification procedures can be classified as being *parametric* or *nonparametric*. In the former, a preliminary model about the system is available, and its finite number of parameters have to be fitted. In the latter, a set of basis functions, capable to describe any input signal, has to be chosen, and the system is probed using certain elements of this—usually infinite—basis (Westwick and Kearney, 2003). Various techniques of system identification are used in deciphering kernels of neural circuitries upstream to retinal ganglion cells.

Most often, nonparametric identification is applied to investigate light-sensitive neural tissue. A large set of white noise based patterns can be used to determine the stimuli to which the investigated neural circuitries are sensitive. The dimensionality of the space of possible white noise stimuli is high, but the circuitries are affected only by a small subset of it. In the simplest case this can be determined by spike-triggered averaging, that is, averaging the stimuli preceding a spike (*reverse correlation*). In this way, the first-order kernel of the system is obtained. If the cell is sensitive to several features, methods of spike-triggered covariance are demanded for their identification (Schwartz et al., 2006) (Samengo and Gollisch, 2013). The Spike-triggered covariance (STC) matrix is also known as the 2nd-order kernel (Wikipedia, 2015). A popular model of circuitries above retinal ganglion cells is the linear-nonlinear-Poisson model, where a linear filter is followed by a static, nonlinear Poisson spike generator (Chichilnisky, 2001).

First Maramelis used white noise analysis for retina research (Marmarelis and Naka, 1972). Spatio-temporal binary (black and white) as well as Gaussian (grayscale or chromatic) white noise was later applied in a series of experiments on the visual tract. By using cathode ray screens, the receptive field (and the spatial part of the kernel), as well as the temporal kernel of salamander retinal ganglion cells were determined at a flickering frequency of 120 Hz (Segev et al., 2006). Gaussian white noise with spatial, temporal and chromatic modulation was applied to determine the spatio-temporal kernel of macaque retinal ganglion cells for red, green and blue colors, and receptive fields at single cone resolution could also be obtained by this method (Chichilnisky, 2001)(Field et al., 2010). Randomly flickering of a spatially uniform illumination was used to investigate adaptation processes (Hosoya et al., 2005) (Baccus and Meister, 2002). Receptive field properties of the visual cortex were investigated using masked white noise stimuli (Nishimoto et al., 2006).

Retinal response to white-noise-like electric stimulation has also been investigated (Fried et al., 2006) (Freeman et al., 2010).

3.5 Light stimuli in virtual environments

In the (visual) virtual reality (VR), most of the visual field is covered by an artificial scene. In the most advanced virtual reality systems, namely the immersive ones, exclusively the virtual world is shown to the user, while cues from the real environment are blocked out. These can be realized either by a head-mounted setup, or by a room, whose inner surface is used as a projection screen (Riener and Harders, 2012). If the projected world depends on actions taken by the organism, the virtual reality system is called closed-loop, otherwise it is open-loop. In closed-loop virtual reality, the organism has some degree of control over the stimulation, which could vary, for example, as a result of body motion (LaValle, 2015).

In cost-effective experiments, stimuli are implemented by printed images lined on cylinders or tunnel walls. For example, tunnels decorated with images containing all spatial frequencies with equal power were also constructed, thus making optic flow estimations distance independent (Linander et al., 2016).

In more flexible solutions, the stimulating environment is created on assembled screen systems. In order to make the illusion of the motion, moving grids (Taylor et al., 2013), or grating with varying period (Dakin et al., 2016) can also be shown on the screens. In the screen ahead of the animal radial gratings have been applied (Saleem et al., 2013). Combined moving gratings and dots are also used to imitate motion (Evans et al., 2011).

4 HARDWARE AND TECHNICAL

Depending on the type of the experiment, the required temporal and spatial resolution of the applied stimuli can vary on several orders of magnitude.

The spatial resolution is rarely limited by pixel number of the light projecting device. For the majority of experiments in visual psychophysics, a 2K resolution (1080×2048) satisfies the demands. If the image is driven into a microscope with a $20\times$ objective and projected on a live specimen, the spatial resolution will mainly be limited by the properties of the microscope objective and that of the perfusion chamber, and not by the pixel count of the projector. For example, if we consider a $20\times$ microscope objective with a resolution of $0.4\ \mu\mathrm{m}$ and a typical field of view having the size of $1 \times 1\mathrm{mm}$, theoretically 2500×2500 pixels would fit into this field. However, the resolution is severely lowered by the perfusion chamber.

For most applications in psychophysics, retina electrophysiology and manipulating membrane potential by optogenetic tools, a satisfactory temporal resolution is offered by personal computer driven projectors (real refresh rate of $60 - 500$ Hz for RGB images) (Packer et al., 2001; PROPixx, 2016; DepthQ 360 DLP Projector, 2016) or LCD monitors (Ghodrati et al., 2015; Wang and Nikolic, 2011) (real refresh rate usually of $60 - 120$ Hz for RGB images). For extremely fine temporal resolution, a series of particular hardware driven by their own software can be applied. By using DMD developer kits with onboard memory, binary image frame rates in the order of 32 kHz can be achieved (Wintech, 2016; Zhu et al., 2012).

4.1 Timing

The time lag of the synchronization voltage signals sent out by GEARS depends on how fast the CPU is notified about the fact that the GPU has displayed a frame, and the time it takes to emit the signal to the USB port. The USB to RS232 conversion can also introduce a delay. On the other hand, any change in the light emitted by the projectors/displays follows the GPU signals with some latency.

We have measured the overall latency by recording both the synchronization voltage signals and the light emitted by a projector (see Figure S1), and found that it was constant. Therefore, it is easy to account for this difference during measurement analysis. Output signals can be handled on the same CPU thread as the one controlling the GPU, or (on multi-core systems) on a separate *busy waiting* thread, which polls the system clock continuously to emit signals with the accuracy of the internal CPU timer.

Note that the amplitude of the synchronization voltage signals depends on the USB to RS232 adapters used. Since most adapters provide output of about $9\mathrm{V}$, a voltage clamp unit could be necessary to finally obtain TTL range $(5\mathrm{V})$ signals. The detailed documentation of the hardware can be downloaded from the Hardware section of the www.gears.vision website.

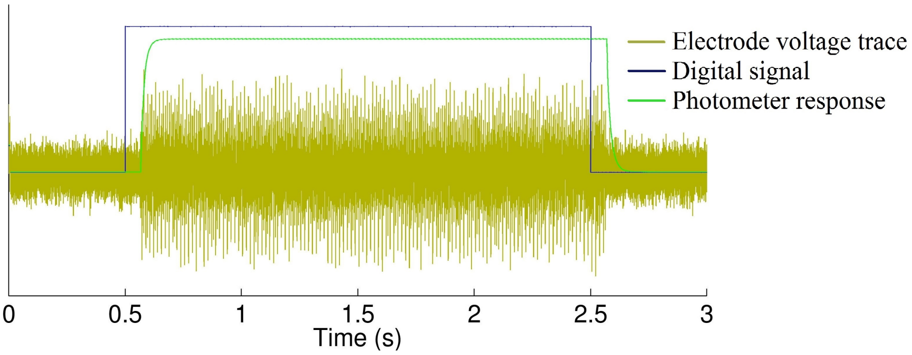

Figure S1: Relative timing of the digital signal sent out by the graphics card to switch on a strong $(2\mathrm{W/cm^2})$ fullfield light stimulus (blue), the noise induced on the illuminated MChS 256MEA 100/30iR-ITO-pr electrodes and the photometer response. Note the cca $70\mathrm{ms}$ delay between the digital signal and the light response. This constant delay, inevitably introduced by the electronics, can easily be handled during data processing.

4.2 Image tearing

A *frame buffer* is a part of GPU memory, reserved for holding an image, that is written to during the rendering process. Video cards maintain a *swap chain* of frame buffers. Only one of them, the *front buffer*, is used to drive the display. The *back buffer* is the one that serves as the output of the GPU pipeline. Rendering a stimulus frame consists of manipulations on the back buffer. This process is invisible, as the back buffer is not shown until a *buffer swap*, when the roles of the two buffers are interchanged. Display monitors refresh the screen regularly, typically at 60 Hz, going line by line, from left to right and from top to bottom of the screen. If the buffer swap happens when the display monitor is in the process of refreshing, half of the pixels will display contents from one buffer, and the other half from the other buffer. If shapes are moving between the frames, this causes them to appear torn along a horizontal line. This *tearing* artifact is unacceptable in retina stimuli. Thus, we have to make sure that buffer swaps only happen between monitor refreshes, i.e. in the *vertical blank interval*. This can be achieved if the graphics card waits for the *vertical sync* signal from the monitor to display the new frame. GEARS enables this hardware option, thus eliminating tearing. However, driver settings may prohibit applications from enacting certain options. These are typically managed from an application provided by the GPU manufacturer (e.g. NVidia Control Panel). If the vertical sync option is forcibly disabled there, GEARS displays a prominent warning on the starting screen. No stimulus sequence can be run, but an error message is issued instead, with instructions on how to change the settings.

4.3 Frame drops

When all buffers of the swap chain are full with rendered images, the program has to wait for a buffer swap. Then, the previous front buffer becomes available as the new back buffer, and the rendering of the next frame can commence. When vertical sync is enabled, buffer swaps are going to happen at the exact frequency of display monitor refreshes. Therefore, the program has this exact amount of time to render a frame of a stimulus. If it fails to do so, it misses the buffer swap, and the image rendered in the previous frame will be displayed for another refresh interval. This *frame drop* is unacceptable in some retina-related experiments.

Many modern video cards offer an option called *triple buffering*. This means that a third buffer is used, but different devices may do so in different ways. One implementation relaxes the time constraints, as it introduces a third buffer in the chain between the back and front buffers. The rendered image is not displayed immediately on a buffer swap, but its buffer becomes this intermediate buffer, and only on the second buffer swap does it become displayed as the front buffer. This means the frame drop only occurs when the rendering time of two consecutive frames exceeds two refresh intervals. This eliminates problems with momentary computer load imbalances, but does not help with a consistently slow rendering process. The other type of triple buffering works with two back buffers, swapping between them as new frames become available, without forcing the program to wait for the vertical sync. On the vertical blank, the latest back buffer is swapped with the front buffer. This method does not offer any feedback to the CPU about the presentation of the frames on the display device, so it is not useful in a high timing precision application like GEARS. As the two triple buffering methods act very differently, with potentially catastrophic consequences, the triple buffering option should be turned off in the hardware control application. We did not use it in our experiments, but did not experience any change in performance or behavior on our test systems when testing with triple buffering enabled, either.

Since image manipulation may require high computational power, its feasibility within a refresh interval depends on the required frame rate, the resolution of the images (both in spatial and frequency domains), and the performance of the graphics card. As GEARS supports a wide range of stimuli with very different computational requirements, where the required performance can strongly depend on parametrization and frame rate, it is not possible to provide a single benchmark that verifies if a computer system will never drop a frame while running GEARS. Stimulus sequences have to be tested individually. Note that the user is always warned if a frame drop happens.

GEARS uses a technique called *shader warm-up* to reduce the occurrence of frame drops. Before launching a stimulus sequence, a single frame of every stimulus in the sequence is rendered, unseen to the user. This forces the graphics driver to perform any optimizations or caching in advance, instead of doing them on a lazy strategy during the stimulus sequence display.

4.4 Gamma compensation

Most commercial displays and projectors are designed to take human visual psychophysics into account. Accordingly, the same intensity variation produces the same differential change in light intensity perception for both high or low values. However, scientific applications assume a strictly linear relationship between the value specified in the software and the resulting change in light intensity. Therefore, the gamma distortion of the device must be compensated. In GEARS, the distortion curve can be specified either as a mathematical function or as tables from manufacturer communications or photometer measurements.

4.5 Color management

GEARS offers RGB color specification. Both ready-made stimuli and **primer** SBCs accept color parameters either as color keywords or intensities on RGB channels. For each display device (*e.g.* various DMD or LCD projectors) spectroradiometric calibration of the individual color channels has to be done separately. If the absorption spectra of the biological light sensors are provided, the relative strength of their excitation for a certain channel of a display device can be computed and displayed.

4.6 Image manipulation basics

Contrast stretching uses a linear transfer function on the pixel intensities, mapping the dynamically measured intensity minimum and maximum to zero and one, which correspond to the extrema of the dynamic range of the display device. Histogram equalization measures the distribution and determines the appropriate transfer function to obtain a uniform distribution of intensities in the unit range. Tone mapping in general may use any transfer function. All of these options are available in GEARS, as described in section S6, using the tone mapping panel on the sequence overview screen (figure S5).

4.7 Implementation of temporal filtering

For temporal filtering, the LTI signal processing approach is preferred over the kernel convolution, since it typically uses a fraction of the storage and the memory access bandwidth, allowing higher frame rates. Any finite-size convolution kernel can be realized by an LTI system, but performance is only improved if a so-called *minimal state representation* with few states is found (De Schutter, 2000). For this job, GEARS contains an implementation of Kung's method (Kung et al., 1983), applying the free matrix library Eigen (Guennebaud, 2013). Depending on the number of the state variables and the characteristics of the convolution kernel, the realization may have some residual error, which is reported to the user. We found that realizations with three state variables are often sufficient, and seven state variables (also supported by GEARS) produces negligible errors for any common function.

5 GRAPHICAL USER INTERFACE (GUI)

GUI performance is of utmost importance in retina physiology and psychophysics experiments, where time and scientist attention are costly resources. A graphical sequence overview is displayed with prominent characteristics plotted over a timeline—so that the scientist conducting the experiment can make a visual verification before launching the actual sequence.

Our GUI has two main screens, and some pop-up dialogs. In the first one (figure S2), which appears when the program is started, one can choose from different stimulus sequences already present in GEARS, organized into a hierarchy. Folders of this hierarchy can have associated measurement configurations, which can be edited using a dialog (figure S3). It is also possible to launch the visual script editor (figure S6) to edit existing sequences and add new ones. After loading a sequence in the first screen, a second screen (figure S4) displays information related to the loaded stimulus sequence and its execution. The top half of the window shows the sequence overview with sample frames, timeline plots of important temporal characteristics (e.g. modulation intensity or synchronization signals), stimulus names and durations, refresh rate, and screen resolution. A stimulus can be selected by clicking the timeline. In the lower half of the screen, the selected stimulus can be explored. A detailed timeline, sample frames, and filter kernel plots are shown. GUI panels for random number export and configuring tone mapping (figure S5) are available.

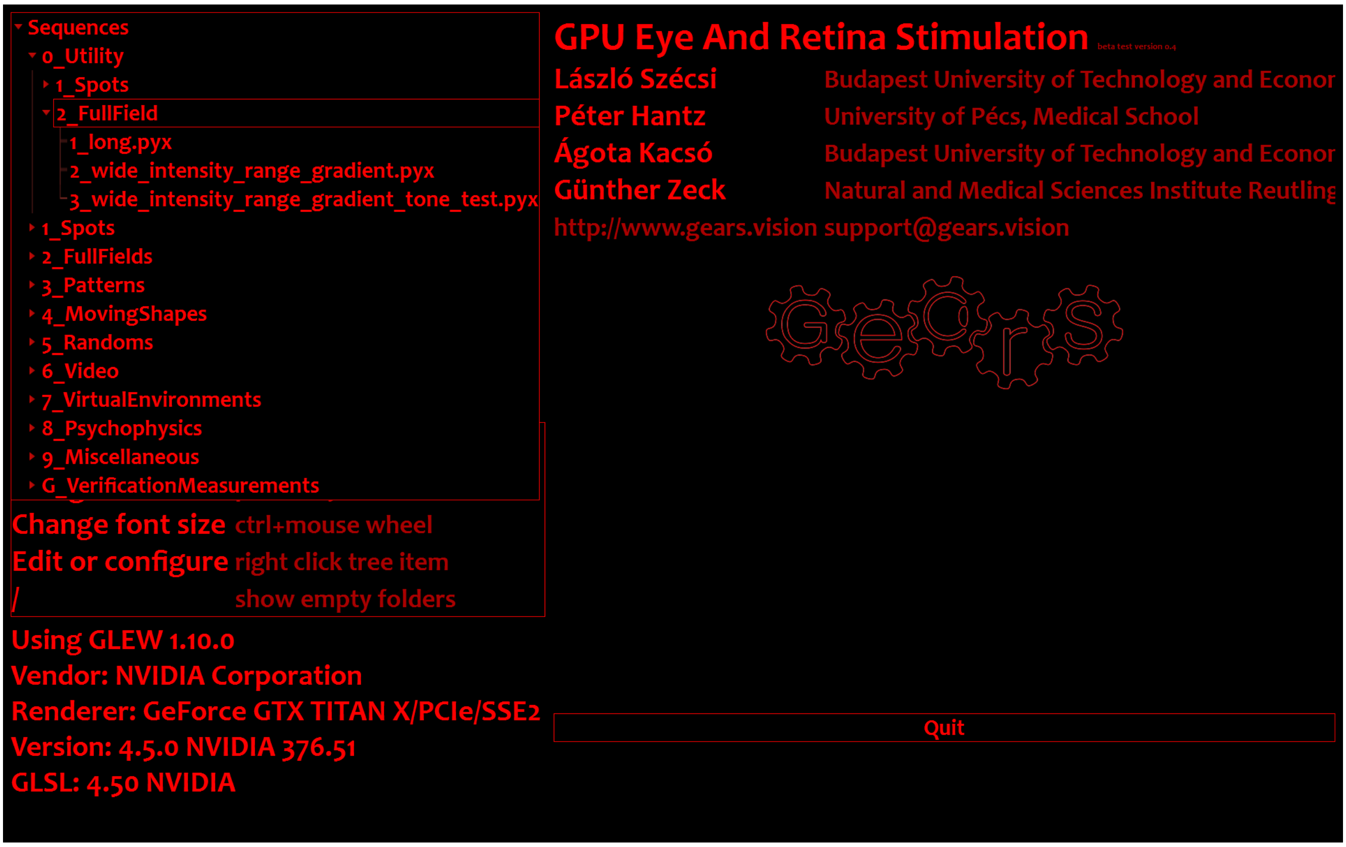

Figure S2: The opening, sequence browser screen, where the user can a navigate the hierarchy of stimulus sequences, open them for execution or editing, and configure the measurement setup.

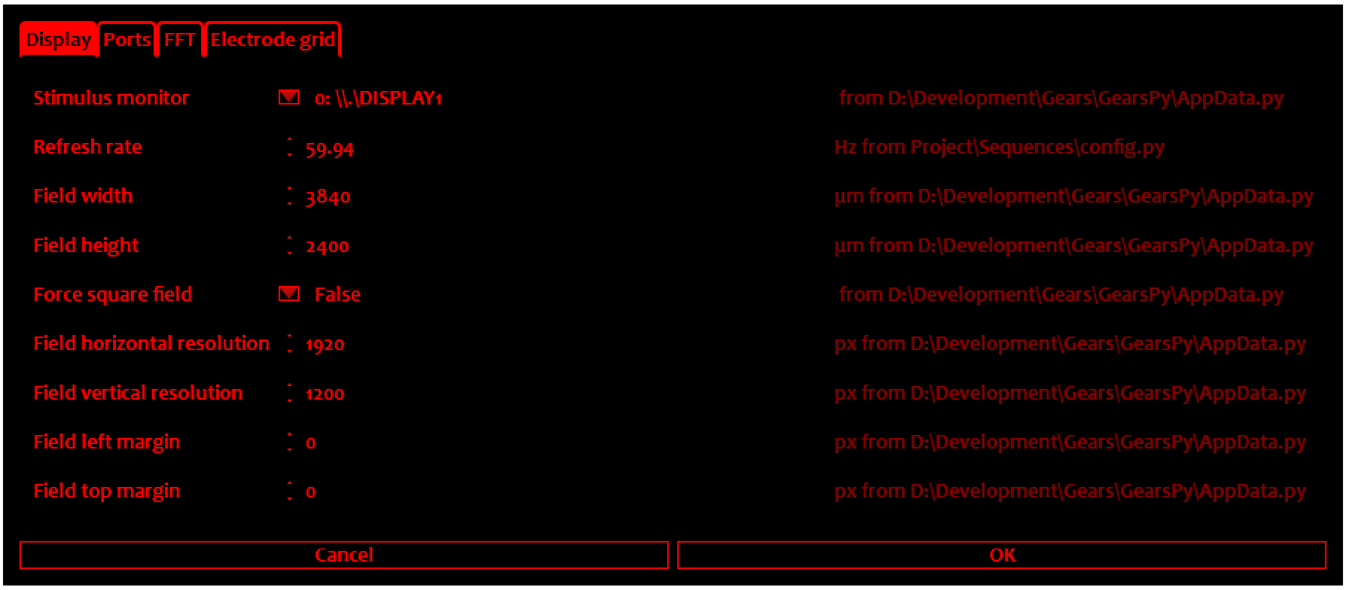

Figure S3: The configuration dialog opened from the sequence browser screen. Display and MEA grid geometry, signals to measurement electronics, and FFT resolution can be specified, or left to inherit settings from the parent sequence folder.

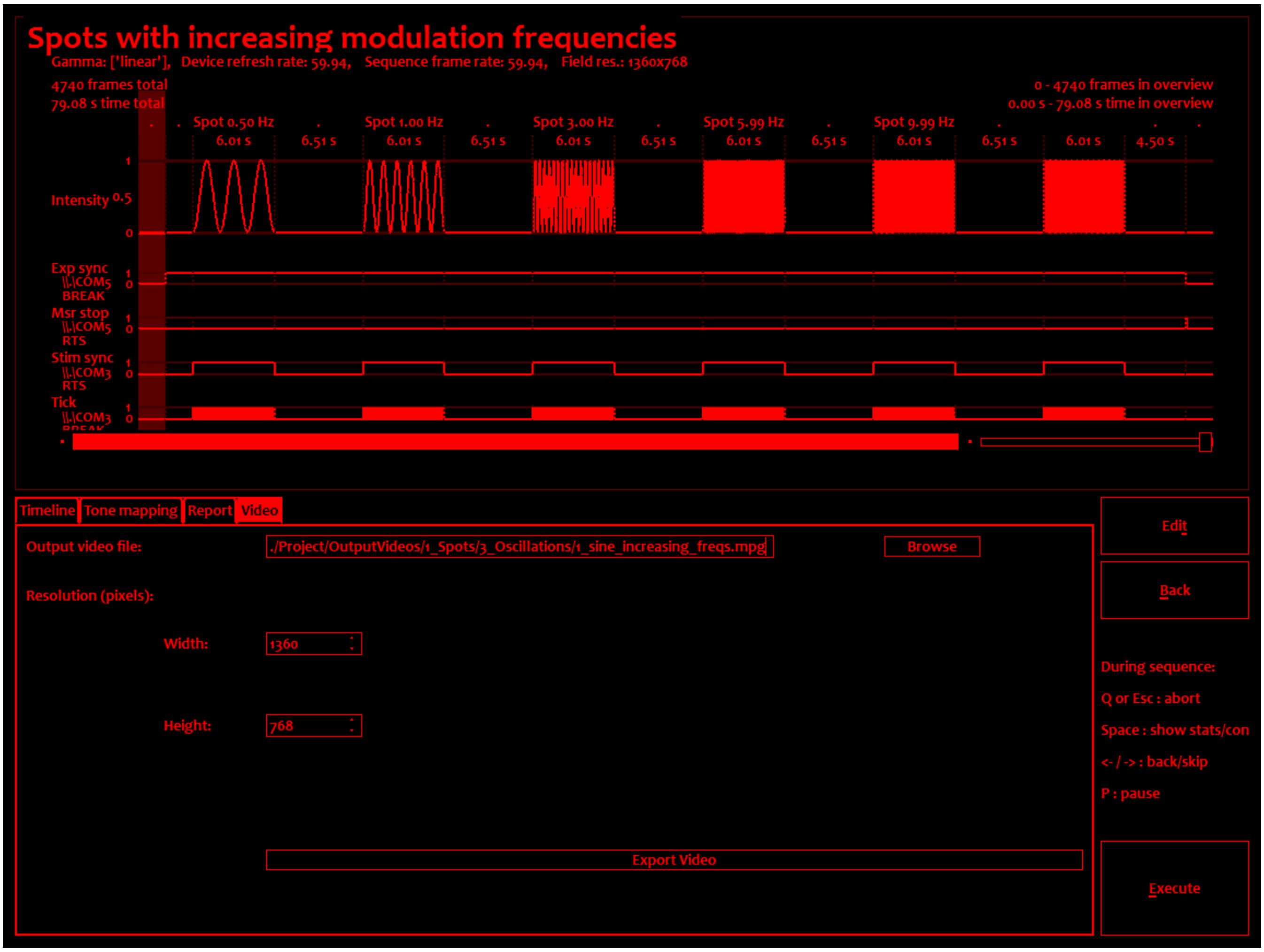

Figure S4: The stimulus sequence overview screen, from where sequence renderings can be launched.

There is also a report window to notify the user of successful experiment execution, or of potential problems like non-responsive data ports or frame drops.

Usually it is detrimental to stimulate the specimen with anything else but the intended visual pattern. The GEARS GUI only uses monochromatic (by default, red) elements on black, to reduce complications when the GUI is accidentally projected onto the specimen.

In addition to stimulus sequence execution, the GUI also offers various management options, including histogram measurement and export of pseudo-random numbers used during the stimulus. Those experiment features that admit graphical editing—like shape outlines—can be edited in-program as well.

Integrated Development Environments (IDE) provide programmers with tools to explore software interfaces without referring to external manuals. The most relevant ones are *code completion* and *call tips*. Code completion can guess the identifier after typing a few characters, but, more importantly, it also offers a list of all possible options available in a certain module. For example, the GEARS IDE lists all **primer** SBCs, when an **primer** component is being added. Call tips display all parameters of functions. In GEARS, we have enhanced this mechanism to display all possible parameters, with their current values, and full descriptions.

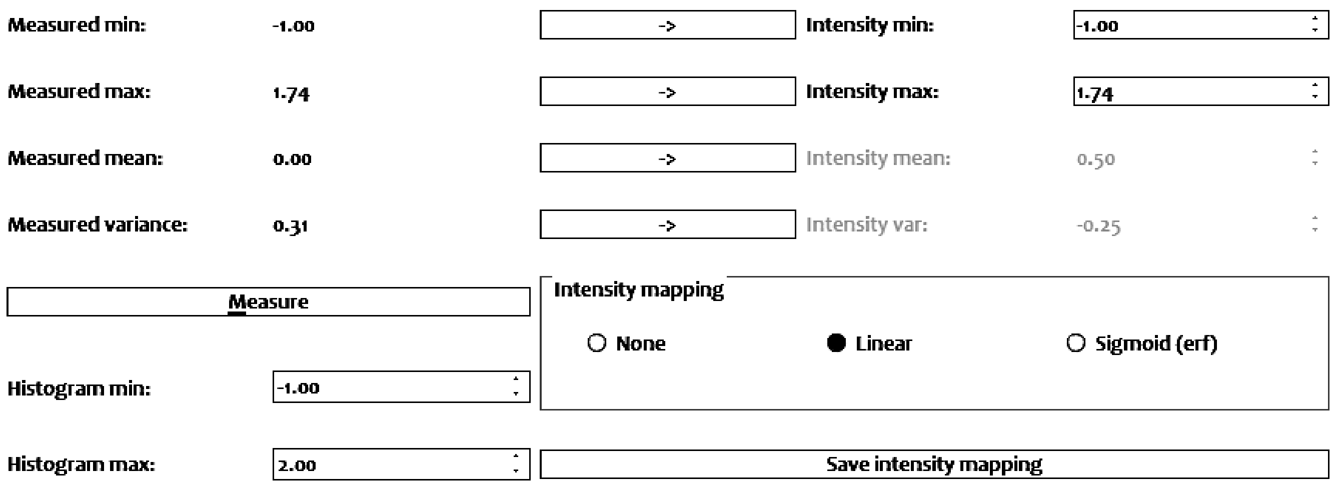

Figure S5: The tone mapping panel. An in-silico measurement can be launched to find the appropriate tone mapping parameters.

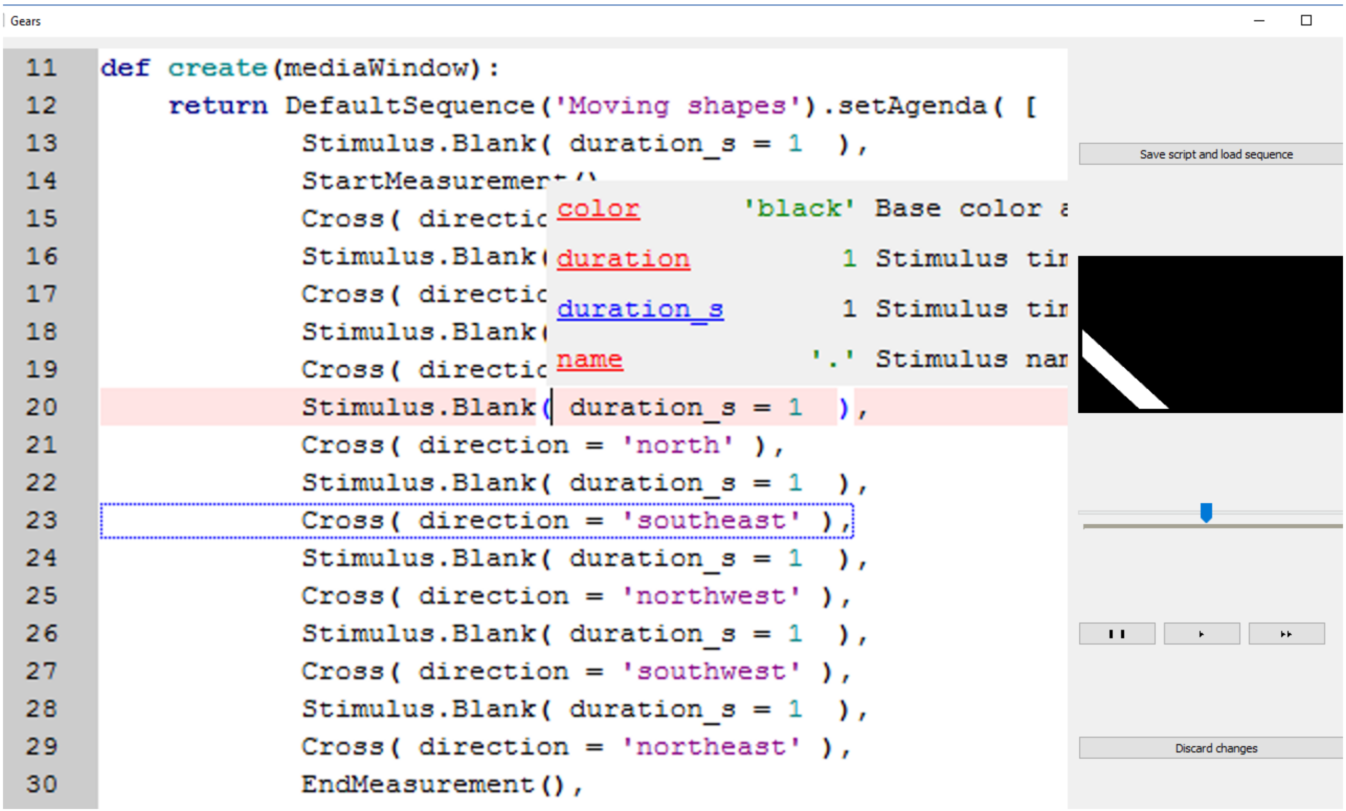

Figure S6: The visually aided scripting interface of GEARS.

Editing stimuli benefits from a what-you-see-is-what-you-get philosophy. Some features like polygon vertices for custom-defined 2D shapes or camera paths in virtual reality can be edited graphically. Where this is not feasible (e.g. when definition of a mathematical function is required), the visual output of a component being edited is instantly displayed, right next to the edited script. In the GEARS visual editor,

the user can play (or seek into) the experiment sequence in a small window next to the edited script, and the parameters corresponding to the currently displayed stimulus are highlighted. On selection, also the explanations, current and default values, and further parameter options are listed.

An alternative for the IDE would be a GUI used not only for stimulus overview and control, but also for stimulus assembly. However, as the example of PsychoPy shows, not even a large community can keep up the effort to provide GUI for all emerging functionality. Furthermore, a script language like Python will always be more powerful than a GUI—or else the complexity of the GUI makes it no better than script editing.

6 VALIDATION EXPERIMENTS

6.1 Linear kernel reconstruction in rodent retina

We recorded the response of the retina to 60Hz-flickering random checkerboards displaying binary (black and white) and uniform (grayscale) white noise. Spike sorting was performed by a custom Matlab program applying the UltraMegaSort2000 package (Hill et al., 2007) Figure S7 shows an 8 minute long sample voltage trace of a retinal ganglion cell, the sorted spikes, as well as the spatial and temporal kernels resulting from the reverse correlation analysis of 48 min recorded data.

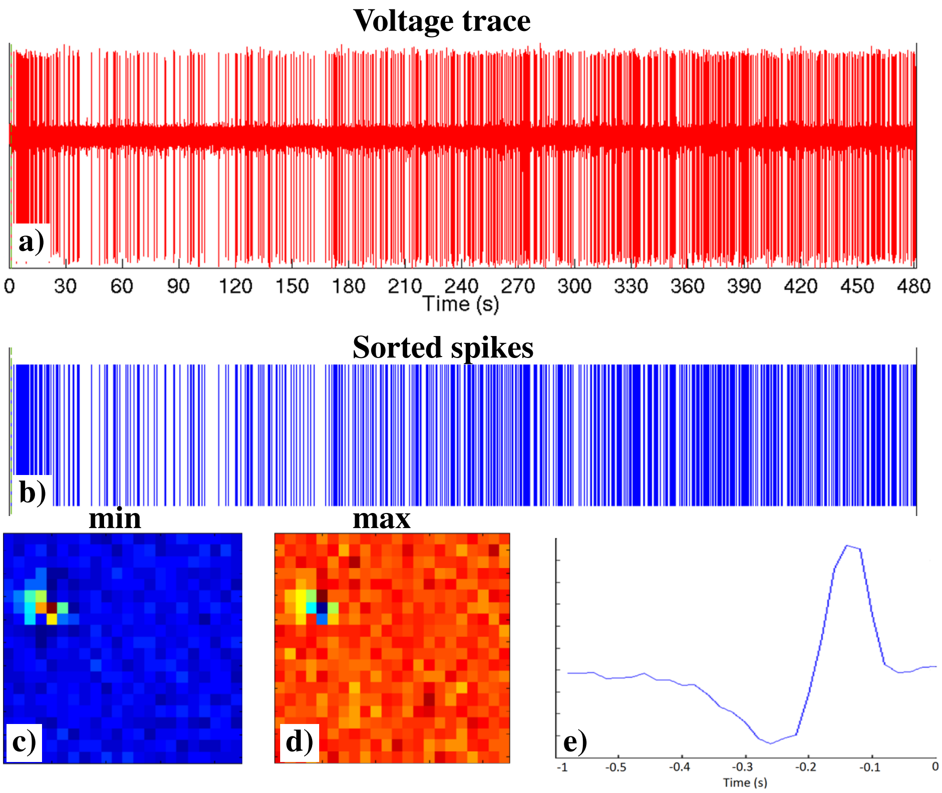

Figure S7: Results of retina stimulation with a randomly flickering checkerboard pattern. a.) The recorded voltage trace at an electrode covering the retinal ganglion cell of interest. b.) The sorted spikes from the voltage trace presented above. c.) and d.) The reconstructed spatial kernel at the time point when its amplitude is maximal and minimal, respectively. e.) The reconstructed temporal kernel.

6.2 OFF-cell response to decreasing illumination

A special stimulus sequence has been elaborated to investigate the response of a subset of OFF-type retinal ganglion cells in mice to decreasing illumination. This stimulus consisted of fullfield illumination which maintains its intensity for two seconds, followed by a uniform decrease to zero in a given time interval. This time interval becomes gradually shorter in subsequent stimuli of the stimulus sequence. Figure S8 shows how the light decrease rate influences the spiking of these cell, which is an important input in building a mathematical model for their behavior. The experiment has been performed in an APB-CPP-NBQX cocktail in Ringer solution.

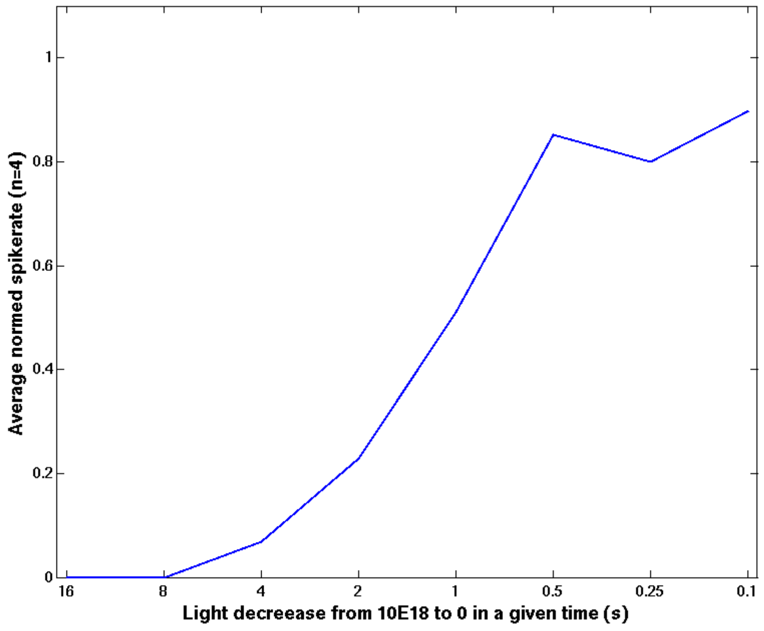

Figure S8: The spike rate of a subset of OFF RGC. The experiment has been carried out in a blocking cocktail (APB-CPP-NBQX)

6.3 Response of mouse retina to full-field flicker stimulation

We recorded the response of the retina to a 70 second stimulus composed of full-field flicker stimulation followed by a frequency modulated chirp stimulus, an amplitude modulated frequency stimulus and moving bars presented in 16 different directions. Spike sorting of the recorded activity was performed by a custom Matlab program applying KlustaKwik spike sorting. Figure S9 shows five repetitions of the stimulus. Robust spiking is detected for each stimulus repetition, as demonstrated in the rasterplot, where each bar represents the occurrence of one ganglion cell spike. Such activity patterns may be used to refine the elaborate ganglion cell classification performed using calcium imaging (Baden et al., 2016).

FURTHER SUPPLEMENTARY FIGURES

Figure S10 shows an example of a non-linear image transformation that can be used for projecting on non-planar surfaces or through custom lens or mirror systems, among others. Figure S11 contains two screenshots of procedural 3D scenes usable as virtual reality environments. The rotating snakes in figure S12 is an example of visual illusion stimuli available in GEARS.

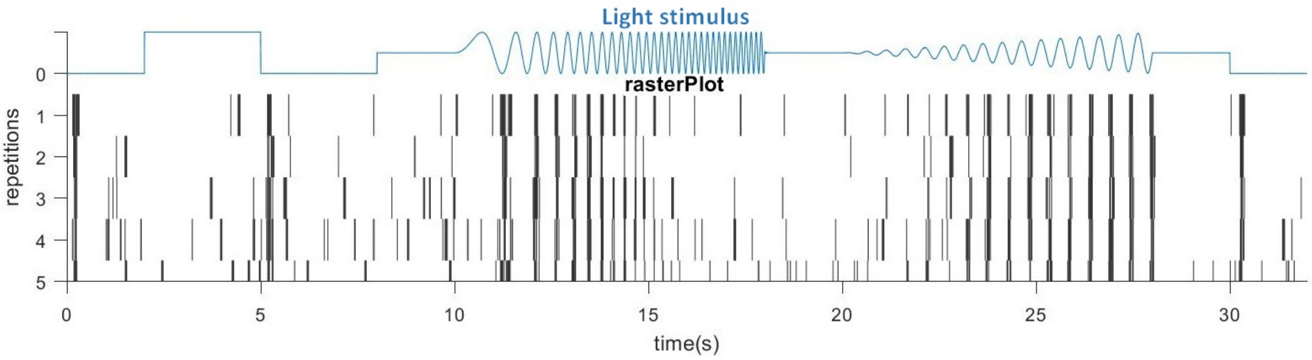

Figure S9: Rasterplot of ganglion cell spikes upon stimulation using a chirp stimulus. Five repetitions of the chirp light stimulus were presented. For each repetition the recorded spikes are presented as ticks in the lower part of the figure.

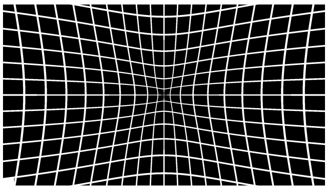

Figure S10: Fisheye distortion of a rectangular grid.

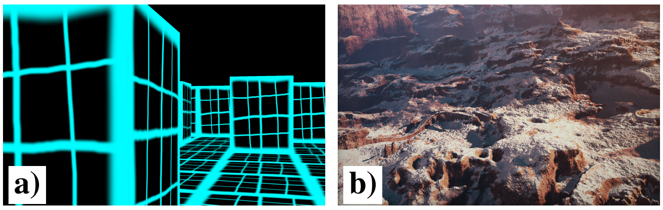

Figure S11: Procedural 3D environments rendered by **a)** forward rendering, and **b)** raycasting (Quilez, 2014).

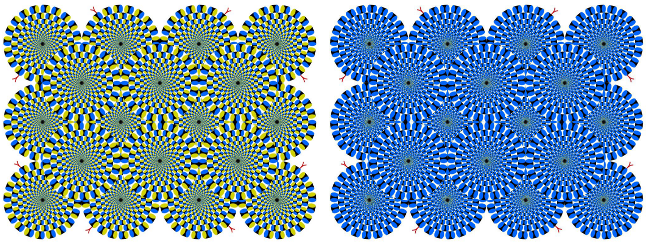

Figure S12: Starting and ending frames of a motion illusion stimulus, where the color of a component is gradually changing to make the illusion disappear.

REFERENCES

Baccus, S. A. and Meister, M. (2002). Fast and slow contrast adaptation in retinal circuitry. *Neuron* 36, 909–919

Baden, T., Berens, P., Franke, K., Rosón, M. R., Bethge, M., and Euler, T. (2016). The functional diversity of retinal ganglion cells in the mouse. *Nature* 529, 345–350

Baker, C. I. (2013). Visual processing in the primate brain. In *Handbook of Psychology, Behavioral Neuroscience*, ed. S. M. Irving B. Weiner, Randy J. Nelson (New Jersey: John Wiley and Sons Inc.), chap. 4. 81–114

Barlow, H. and Levick, W. R. (1965). The mechanism of directionally selective units in rabbit's retina. *The Journal of physiology* 178, 477

Bellard, F., Niedermayer, M., et al. (2016). Ffmpeg. *http://ffmpeg.org*

Benoit, A., Caplier, A., Durette, B., and Hérault, J. (2010). Using human visual system modeling for bio-inspired low level image processing. *Computer vision and Image understanding* 114, 758–773

Chichilnisky, E. (2001). A simple white noise analysis of neuronal light responses. *Network: Computation in Neural Systems* 12, 199–213

Clark, D. A., Benichou, R., Meister, M., and da Silveira, R. A. (2013). Dynamical adaptation in photoreceptors. *PLOS Comput Biol* 9, e1003289

Dakin, R., Fellows, T. K., and Altshuler, D. L. (2016). Visual guidance of forward flight in hummingbirds reveals control based on image features instead of pattern velocity. *Proceedings of the National Academy of Sciences* , 201603221

Daugman, J. G. (1980). Two-dimensional spectral analysis of cortical receptive field profiles. *Vision research* 20, 847–856

De Schutter, B. (2000). Minimal state-space realization in linear system theory: an overview. *Journal of Computational and Applied Mathematics* 121, 331–354

Demb, J. B., Haarsma, L., Freed, M. A., and Sterling, P. (1999). Functional circuitry of the retinal ganglion cell's nonlinear receptive field. *The Journal of Neuroscience* 19, 9756–9767

Demb, J. B. and Singer, J. H. (2015). Functional circuitry of the retina. *Annual Review of Vision Science* 1, 263–289

DepthQ 360 DLP Projector (2016). Cambridge research systems. `http://www.crsltd.com/tools-for-vision-science/displays/depthq-360-dlp-projector/`

Dowling, J. E. (1987). *The retina: an approachable part of the brain* (Harvard University Press)

Enroth-Cugell, C. and Robson, J. G. (1966). The contrast sensitivity of retinal ganglion cells of the cat. *The Journal of physiology* 187, 517–552

Evans, O., Paulk, A. C., and van Swinderen, B. (2011). An automated paradigm for drosophila visual psychophysics. *PloS one* 6, e21619

Farrow, K., Teixeira, M., Szikra, T., Viney, T. J., Balint, K., Yonehara, K., et al. (2013). Ambient illumination toggles a neuronal circuit switch in the retina and visual perception at cone threshold. *Neuron* 78, 325–338

Field, G. D., Gauthier, J. L., Sher, A., Greschner, M., Machado, T. A., Jepson, L. H., et al. (2010). Functional connectivity in the retina at the resolution of photoreceptors. *Nature* 467, 673–677

Freeman, D. K., Rizzo III, J. F., and Fried, S. (2010). Electric stimulation with sinusoids and white noise for neural prostheses. *Frontiers in neuroscience* 4, 1

Fried, S. I., Hsueh, H.-A., and Werblin, F. S. (2006). A method for generating precise temporal patterns of retinal spiking using prosthetic stimulation. *Journal of neurophysiology* 95, 970–978

Ghodrati, M., Morris, A. P., and Price, N. S. C. (2015). The (un) suitability of modern liquid crystal displays (lcds) for vision research. *Frontiers in psychology* 6

Guennebaud, G. (2013). Eigen. *http://eigen.tuxfamily.org*

Herault, J., Cristobal, G., Perrinet, L., and Keil, M. S. (2015). *Biologically Inspired Computer Vision: Fundamentals and Applications* (John Wiley & Sons)

Hill, D., Kleinfeld, D., and Mehta, S. (2007). Spike sorting. *Observed Brain Dynamics by PP Mitra and H. Bokil. Oxford Press* 9, 257–270

Hoggarth, A., McLaughlin, A. J., Ronellenfitch, K., Trenholm, S., Vasandani, R., Sethuramanujam, S., et al. (2015). Specific wiring of distinct amacrine cells in the directionally selective retinal circuit permits independent coding of direction and size. *Neuron* 86, 276–291

Hosoya, T., Baccus, S. A., and Meister, M. (2005). Dynamic predictive coding by the retina. *Nature* 436, 71–77

Hubel, D. H., Wensveen, J., and Wick, B. (1995). *Eye, brain, and vision* (Scientific American Library New York)

Hubel, D. H. and Wiesel, T. N. (1959). Receptive fields of single neurones in the cat's striate cortex. *The Journal of physiology* 148, 574–591

Ichinose, T., Fyk-Kolodziej, B., and Cohn, J. (2014). Roles of on cone bipolar cell subtypes in temporal coding in the mouse retina. *The Journal of Neuroscience* 34, 8761–8771

Joselevitch, C. and Kamermans, M. (2009). Retinal parallel pathways: seeing with our inner fish. *Vision research* 49, 943–959

Kuffler, S. W. (1953). Discharge patterns and functional organization of mammalian retina. *Journal of neurophysiology* 16, 37–68

Kung, S.-Y., Arun, K. S., and Rao, D. B. (1983). State-space and singular-value decomposition-based approximation methods for the harmonic retrieval problem. *JOSA* 73, 1799–1811

LaValle, S. M. (2015). *Virtual reality* (University of Illinois)

Lawless, H. T. (2013). *Quantitative sensory analysis: Psychophysics, models and intelligent design* (John Wiley & Sons)

Linander, N., Baird, E., and Dacke, M. (2016). Bumblebee flight performance in environments of different proximity. *Journal of Comparative Physiology A* 202, 97–103

Livingstone, M. S. and Conway, B. R. (2003). Substructure of direction-selective receptive fields in macaque v1. *Journal of Neurophysiology* 89, 2743–2759

Lu, Z.-L. and Dosher, B. (2013). *Visual psychophysics: From laboratory to theory* (MIT Press)

Marmarelis, P. Z. and Naka, K.-I. (1972). White-noise analysis of a neuron chain: an application of the wiener theory. *Science* 175, 1276–1278

Medathati, N. K., Neumann, H., Masson, G. S., and Kornprobst, P. (2016). Bio-inspired computer vision: Towards a synergistic approach of artificial and biological vision. *Computer Vision and Image Understanding*

Michaelbach (2016). Optical illusions and visual phenomena. `http://www.michaelbach.de/ot/`. Accessed: 2016-02-25

Münch, T. A., Da Silveira, R. A., Siegert, S., Viney, T. J., Awatramani, G. B., and Roska, B. (2009). Approach sensitivity in the retina processed by a multifunctional neural circuit. *Nature neuroscience* 12, 1308–1316

Nishimoto, S., Ishida, T., and Ohzawa, I. (2006). Receptive field properties of neurons in the early visual cortex revealed by local spectral reverse correlation. *The Journal of neuroscience* 26, 3269–3280

Ölveczky, B. P., Baccus, S. A., and Meister, M. (2003). Segregation of object and background motion in the retina. *Nature* 423, 401–408

Packer, A. M., Roska, B., and Häusser, M. (2013). Targeting neurons and photons for optogenetics. *Nature neuroscience* 16, 805–815

Packer, O., Diller, L. C., Verweij, J., Lee, B. B., Pokorny, J., Williams, D. R., et al. (2001). Characterization and use of a digital light projector for vision research. *Vision research* 41, 427–439

Pelli, D. G. and Farell, B. (1995). Psychophysical methods. *Handbook of optics* 1, 29–1

PROPixx (2016). *Projector optimized for vision researchers*. VPixx Technologies Inc.

Quilez, I. (2014). Canyon. `https://www.shadertoy.com/view/MdBGzG`. Accessed: 2016-02-25

Riener, R. and Harders, M. (2012). *VR for medical training* (Springer)

Rodieck, R. W. (1965). Quantitative analysis of cat retinal ganglion cell response to visual stimuli. *Vision research* 5, 583–601

Sakai, H. M., Ken-Ichi, N., and Korenberg, M. J. (1988). White-noise analysis in visual neuroscience. *Visual neuroscience* 1, 287–296

Saleem, A. B., Ayaz, A., Jeffery, K. J., Harris, K. D., and Carandini, M. (2013). Integration of visual motion and locomotion in mouse visual cortex. *Nature neuroscience* 16, 1864–1869

Samengo, I. and Gollisch, T. (2013). Spike-triggered covariance: geometric proof, symmetry properties, and extension beyond gaussian stimuli. *Journal of computational neuroscience* 34, 137–161

Schwartz, G., Taylor, S., Fisher, C., Harris, R., and Berry, M. J. (2007). Synchronized firing among retinal ganglion cells signals motion reversal. *Neuron* 55, 958–969

Schwartz, O., Pillow, J. W., Rust, N. C., and Simoncelli, E. P. (2006). Spike-triggered neural characterization. *Journal of Vision* 6, 13–13

Segev, R., Puchalla, J., and Berry, M. J. (2006). Functional organization of ganglion cells in the salamander retina. *Journal of neurophysiology* 95, 2277–2292

Shapley, R. (2009). Linear and nonlinear systems analysis of the visual system: Why does it seem so linear?: A review dedicated to the memory of Henk Spekreijse. *Vision research* 49, 907–921

Shapley, R. and Victor, J. (1978). The effect of contrast on the transfer properties of cat retinal ganglion cells. *The Journal of physiology* 285, 275

Smirnakis, S. M., Berry, M. J., Warland, D. K., Bialek, W., Meister, M., et al. (1997). Adaptation of retinal processing to image contrast and spatial scale. *Nature* 386, 69–73

Snowden, R., Snowden, R. J., Thompson, P., and Troscianko, T. (2012). *Basic vision: an introduction to visual perception* (Oxford University Press)

Taylor, G. J., Luu, T., Ball, D., and Srinivasan, M. V. (2013). Vision and air flow combine to streamline flying honeybees. *Scientific reports* 3

Troncoso, X. G., Macknik, S. L., and Martinez-Conde, S. (2011). Vision's first steps: Anatomy, physiology, and perception in the retina, lateral geniculate nucleus, and early visual cortical areas. In *Visual Prosthetics* (Springer). 23–57

Van de Grind, W., Grüsser, O.-J., and Lunkenheimer, H.-U. (1973). Temporal transfer properties of the afferent visual system psychophysical, neurophysiological and theoretical investigations. In *Central Processing of Visual Information A: Integrative Functions and Comparative Data* (Springer). 431–573

Wang, P. and Nikolic, D. (2011). An lcd monitor with sufficiently precise timing for research in vision. *Frontiers in human neuroscience* 5, 85

Warrant, E., Oskarsson, M., and Malm, H. (2014). The remarkable visual abilities of nocturnal insects: neural principles and bioinspired night-vision algorithms. *Proceedings of the IEEE* 102, 1411–1426

Wässle, H. (2004). Parallel processing in the mammalian retina. *Nature Reviews Neuroscience* 5, 747–757

Westwick, D. T. and Kearney, R. E. (2003). *Identification of nonlinear physiological systems*, vol. 7 (John Wiley & Sons)

Wikipedia (2015). Spike-triggered covariance. `https://en.wikipedia.org/wiki/Spike-triggered_covariance`. Accessed: 2016-02-25

Wintech (2016). *W4100 high value developer kit*. Texas Instruments

Zhang, Y., Kim, I.-J., Sanes, J. R., and Meister, M. (2012). The most numerous ganglion cell type of the mouse retina is a selective feature detector. *Proceedings of the National Academy of Sciences* 109, E2391–E2398

Zhu, P., Fajardo, O., Shum, J., Schärer, Y.-P. Z., and Friedrich, R. W. (2012). High-resolution optical control of spatiotemporal neuronal activity patterns in zebrafish using a digital micromirror device. *Nature protocols* 7, 1410–1425
